# Spin-selection-favored peroxymonosulfate activation on reaction-induced Co–N_1_O_2_ sites enables ^1^O_2_-biased yet radical-parallel oxidation

**DOI:** 10.1093/nsr/nwag334

**Published:** 2026-06-02

**Authors:** Junlei Zhang, Guojia Yu, Qingqing Zhang, Haoran Wei, Wei Zhao, Shijie Li, Zhiyao Duan, Sihui Zhan

**Affiliations:** School of Materials Science and Engineering, Northwestern Polytechnical University, Xi’an 710072, China; School of Materials Science and Engineering, Northwestern Polytechnical University, Xi’an 710072, China; School of Materials Science and Engineering, Northwestern Polytechnical University, Xi’an 710072, China; School of Materials Science and Engineering, Northwestern Polytechnical University, Xi’an 710072, China; School of Ecology and Environmental Science, Institute for Ecological Research and Pollution Control of Plateau Lakes, Yunnan University, Kunming 650500, China; National Engineering Research Center for Marine Aquaculture, Zhejiang Key Laboratory of Petrochemical Environmental Pollution Control, Zhejiang Ocean University, Zhoushan 316000, China; School of Materials Science and Engineering, Northwestern Polytechnical University, Xi’an 710072, China; School of Environmental Science & Engineering, Tianjin University, Tianjin 300350, China

**Keywords:** single-atom Co catalyst, reaction-induced coordination evolution, spin-state-mediated peroxymonosulfate activation, selective ^1^O_2_ generation, emerging contaminant removal

## Abstract

Transformations in metal coordination environments, particularly their impact on spin-state transitions, remain underexplored in peroxymonosulfate (PMS)-based advanced oxidation processes (AOPs). In this study, we synthesize a Co_1_/C_3_N_5_ single-atom catalyst using a coordination–recrystallization strategy followed by argon pyrolysis. The Co–N_2_O_1_ sites evolve to Co–N_1_O_2_ upon PMS activation, enabling spin-selection-favored activation and efficient pollutant degradation. The evolved site enables spin-selection-favored PMS activation that co-generates sulfate radicals (·SO_4_⁻) and singlet oxygen (^1^O_2_), operating in a ^1^O_2_-biased yet radical-parallel regime. Within this regime, Co_1_/C_3_N_5_ achieves a 5.3-fold increase in the pseudo-first-order rate constant (*k*_app_) for oxytetracycline degradation relative to pristine C_3_N_5_ under identical conditions, and it consistently outperforms more than 30 state-of-the-art PMS-based catalysts. The catalyst further exhibits broad-spectrum activity toward structurally diverse antibiotics and dyes, while maintaining durability in matrix-rich water and under continuous-flow operation. Overall, this work links reaction-induced single-site evolution to spin-aware oxygen transfer, providing concise design guidance for selective and robust PMS-activated AOP catalysts.

## Teaser

Single-atom cobalt catalysts evolve their coordination environment during reaction, biasing peroxymonosulfate activation toward singlet oxygen via spin selection, enabling efficient pollutant degradation with cooperating radical pathways.

## INTRODUCTION

Emerging contaminants (ECs), including antibiotics, industrial dyes, and other trace organics, are increasingly detected in aquatic environments, raising serious concerns due to their persistence, toxicity, and potential for bioaccumulation, which threaten both human health and ecological stability [1–3]. Among the diverse portfolio of water treatment technologies, peroxymonosulfate-based advanced oxidation processes (PMS-AOPs) have attracted substantial attention as a highly adaptable and efficient platform for EC removal [4,5]. These systems offer unique advantages, such as the controllable generation of reactive species via radical and non-radical pathways, robust performance across a broad pH range, and the tunability of catalyst electronic structures to accelerate degradation kinetics and enhance treatment selectivity [6]. A central and emerging frontier in PMS-AOP development lies in the rational design of efficient, sustainable, and environmentally benign activators that can precisely steer the formation of desired reactive species for targeted and high-performance contaminant degradation [7–10].

Single-atom catalysts (SACs) have emerged as a compelling frontier in PMS-AOP research, owing to their maximized atomic utilization, well-defined active sites, and unparalleled tunability in coordination environments and electronic structures [5,6,11]. In recent years, significant efforts have been devoted to advancing both the catalytic performance and mechanistic understanding of SACs in PMS activation. On the performance front, a suite of rational optimization strategies has been proposed (Fig. S1a), including modulation of the coordination number and type of metal centers to tune the electronic environment of the active sites (for example, Fe–N_4+1_, Co–N_3_, CuN_3_–S/P) [12–14], and compositing with metal or metal oxide nanocrystal (for example, Fe–Ni, Co–O–Zn, Co_3_O_4_@Co_1_/C_3_N_5_, Co_SAs-NCs_/CN/TiO_2_) [10,15–17]. These strategies aim to refine the local microenvironment of single-atom sites, thereby enhancing PMS adsorption, activation efficiency, and selectivity toward desired reactive species, ultimately promoting efficient and selective degradation of ECs.

Concurrently, mechanistic investigations combining detailed experimental characterizations with density functional theory (DFT) calculations have been extensively employed to elucidate the fundamental pathways and electronic origins of PMS activation at SAC sites, along with the subsequent generation of reactive species through radical and/or non-radical pathways [18]. The local structure of the active center in SACs—including coordination number, identity of coordinating atoms, and electronic states—plays a pivotal role in determining PMS activation performance [19,20]. However, due to their high surface energy, single-atom sites are intrinsically susceptible to structural perturbation or even dynamic reconstruction under harsh redox environments such as PMS activation [21]. Although previous studies have shown that the coordination environment of single-atom centers can regulate their electronic structure [22], spin state [23], and catalytic behavior, these analyses are predominantly based on static active-site models [24–27], in which the coordination configuration is assumed to remain unchanged during reaction. Such a framework is useful for establishing structure–activity relationships, but it may overlook the possibility that the active site undergoes adaptive coordination evolution under real operating conditions. In this regard, the key distinction of the present work is that we identify a reaction-induced transformation of the Co coordination environment from Co–N_2_O_1_ to Co–N_1_O_2_ during PMS activation, as supported by both theoretical calculations and spectroscopic evidence. This result suggests that spin-selective PMS activation in single-atom systems may not be governed solely by the initial coordination structure, but also by the dynamic evolution of the active site under reaction conditions. Therefore, beyond conventional static coordination engineering, this work introduces a dynamic coordination-evolution perspective for understanding and regulating reactive oxygen species (ROS) generation pathways in PMS-based advanced oxidation systems.

In this work, we synthesize cobalt SACs anchored on nitrogen-rich carbon nitride (Co_1_/C_3_N_5_) via a coordination–crystallization route followed by argon-flow pyrolysis. Pre-/post-reaction spectroscopies indicate a reaction-induced evolution of the Co coordination environment from Co–N_2_O_1_ to Co–N_1_O_2_, while the bulk phase and surface chemical signatures remain largely unchanged (Fig. S1b). Under matched conditions, Co_1_/C_3_N_5_ exhibits higher pseudo-first-order rates than pristine C_3_N_5_ and maintains activity across pH 3–10, diverse water matrices, and repeated cycles; representative benchmarks are shown in the main text and harmonized comparisons are summarized in Table S2. A composite evidence set—dose-dependent scavengers and spin-trapping EPR—supports a ^1^O_2_-biased yet radical-parallel regime, and DFT provides a spin-selection-favored rationale centered at the Co–N_1_O_2_ moiety. Collectively, these results link reaction-induced single-site evolution to oxygen-transfer selectivity in PMS activation and outline design principles for selective, robust AOP catalysts.

## RESULTS AND DISCUSSION

### Synthesis and structural characterization of Co_1_/C_3_N_5_

**Figure 1.**
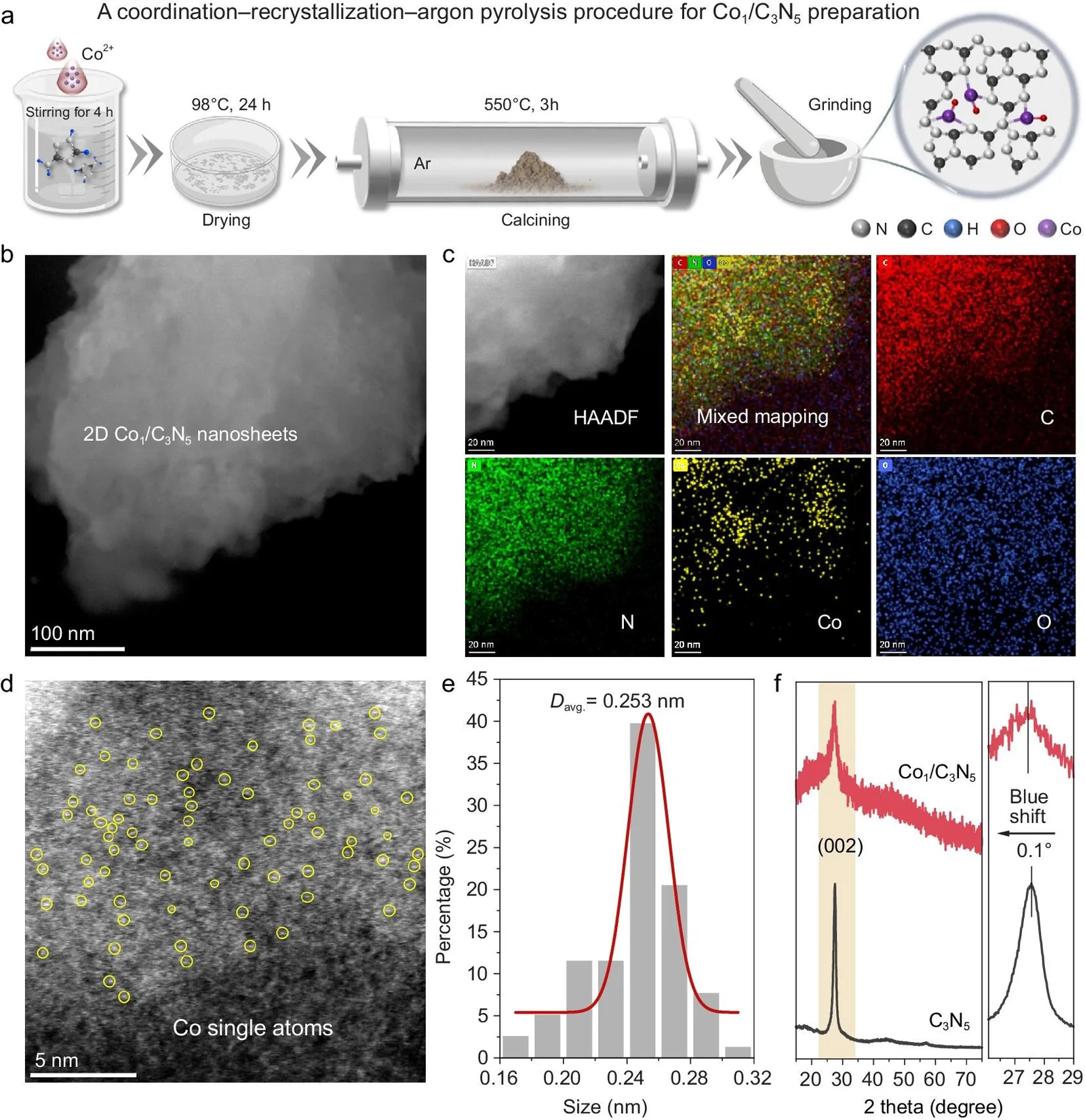
(a) Schematic synthesis of Co_1_/C_3_N_5_ via coordination–crystallization and flow-argon pyrolysis. (b) High-angle-annular-dark-field scanning transmission electron microscopy (AC-HAADF-STEM) image of nanosheets. (c) EDS elemental maps (C, N, O, Co). (d) Aberration-corrected HAADF-STEM (AC-HAADF-STEM) highlighting isolated Co atoms (circles). (e) Apparent bright-spot size distribution extracted from (d). (f) XRD patterns of Co_1_/C_3_N_5_ and pristine C_3_N_5_; the loading-dependent left shift of the (002) reflection (Fig. S2) indicates interlayer expansion upon Co incorporation.

Cobalt SACs anchored on nitrogen-rich carbon nitride (Co_1_/C_3_N_5_) was synthesized through a coordination–recrystallization route followed by flow-argon pyrolysis (Fig. 1a). Notably, this strategy is not restricted to Co incorporation, but can also be extended to the preparation of other C_3_N_5_-based transition-metal SACs with promising catalytic performance, demonstrating good synthetic versatility [28]. Compared with conventional synthetic approaches, this method avoids the use of sophisticated precision equipment, provides a relatively high product yield (∼60%), and is compatible with batch production, thereby offering clear advantages in terms of cost control and scale-up feasibility. Moreover, the good stability and reproducibility of this process suggest considerable potential for the large-scale manufacturing and prospective industrial application of SACs. Unless otherwise specified, Co_1_/C_3_N_5_ refers to the sample with the optimized Co loading (Co_1_/C_3_N_5_-2).

Aberration-corrected high-angle annular dark-field scanning transmission electron microscopy (HAADF-STEM) reveals that Co_1_/C_3_N_5_ adopts an ultrathin, two-dimensional nanosheet morphology (Fig. 1b). Elemental mapping by energy-dispersive X-ray spectroscopy (EDS; Fig. 1c) confirms the homogeneous distribution of Co, C, N, and O throughout the material, underscoring the efficacy of our synthetic approach in achieving intimate dispersion of cobalt species. Critically, atomic-resolution HAADF-STEM images (Fig. 1d) disclose the presence of numerous isolated cobalt atoms, unambiguously identified as bright atomic-scale features (highlighted by yellow circles). These observations indicate atomic dispersion and intimate anchoring of Co within the C_3_N_5_ matrix. The measured bright-spot size of the Co species (∼0.253 nm; Fig. 1e) is consistent with the expected size of single Co atoms, further corroborating the atomically dispersed nature of the active sites.

Powder X-ray diffraction (XRD) analysis was employed to probe the crystalline characteristics of the catalysts. Pristine C_3_N_5_ exhibits a broad diffraction peak centerd at 27.5°, corresponding to the (002) plane of the graphitic framework (Fig. 1f), in agreement with previous reports [9,10]. The XRD pattern of Co_1_/C_3_N_5_ displays a single broad reflection at 27.4°, characteristic of the C_3_N_5_ (002) plane, with no detectable diffraction peaks attributable to cobalt oxides, metallic cobalt, or cobalt-based nanoparticles. Compared to pure C_3_N_5_, the Co_1_/C_3_N_5_ (002) diffraction peak exhibits a blue shift of 0.1°, indicating that the introduction of Co atoms induces lattice expansion. These results suggest that cobalt is incorporated without the formation of crystalline Co aggregates, in line with our microscopic observations. Notably, systematic XRD analysis across the Co-loading series (Fig. S2) reveal a progressive leftward shift of the (002) reflection with increasing cobalt content. This shift is ascribed to the intercalation or coordination of Co atoms within the C_3_N_5_ framework, leading to an expansion of the interlayer spacing. Such lattice modulation further substantiates the successful atomic-level integration of Co into the C_3_N_5_ structure.

Surface chemistry and local coordination were probed by X-ray photoelectron spectroscopy (XPS) and X-ray absorption spectroscopy [29]. The XPS survey spectrum (Fig. S3) confirms the presence of C, N, O, and Co elements, attesting to the successful incorporation of cobalt into the C_3_N_5_ matrix. High-resolution C 1s spectra (Fig. S4) could be deconvoluted into three distinct components at 284.8, 286.2, and 398.1 eV, corresponding to C–C/C=C, C–O, and N–C=N functionalities, respectively—in agreement with canonical features of C_3_N_5_ frameworks [9,10].

The N 1s spectra (Fig. S5a and b) reveal three primary nitrogen species: pyridinic N (∼398.5 eV), pyrrolic N (∼399.8 eV), and graphitic N (∼400.8 eV). Notably, upon cobalt incorporation, subtle shifts in the binding energies of pyrrolic N and graphitic N were observed (to ∼400.0 and ∼400.9 eV, respectively), indicative of electronic interactions between Co atoms and the nitrogen moieties. Critically, an additional peak emerged at 399.1 eV, attributable to Co–N coordination bonds [30]. The relative intensity of this Co–N feature increased systematically with higher cobalt loading, corroborating the formation of Co–N coordination structures within the C_3_N_5_ lattice (Fig. S5c). Complementary O 1s spectra (Fig. S6) were deconvoluted into three components centered at 533.3, 532.0, and 530.7 eV, corresponding to adsorbed OH, adsorbed H_2_O, and Co–O bonds, respectively. The presence and growth of the Co–O component with increasing Co content suggest the coexistence of Co–O coordination motifs, in line with literature precedents [31,32].

**Figure 2.**
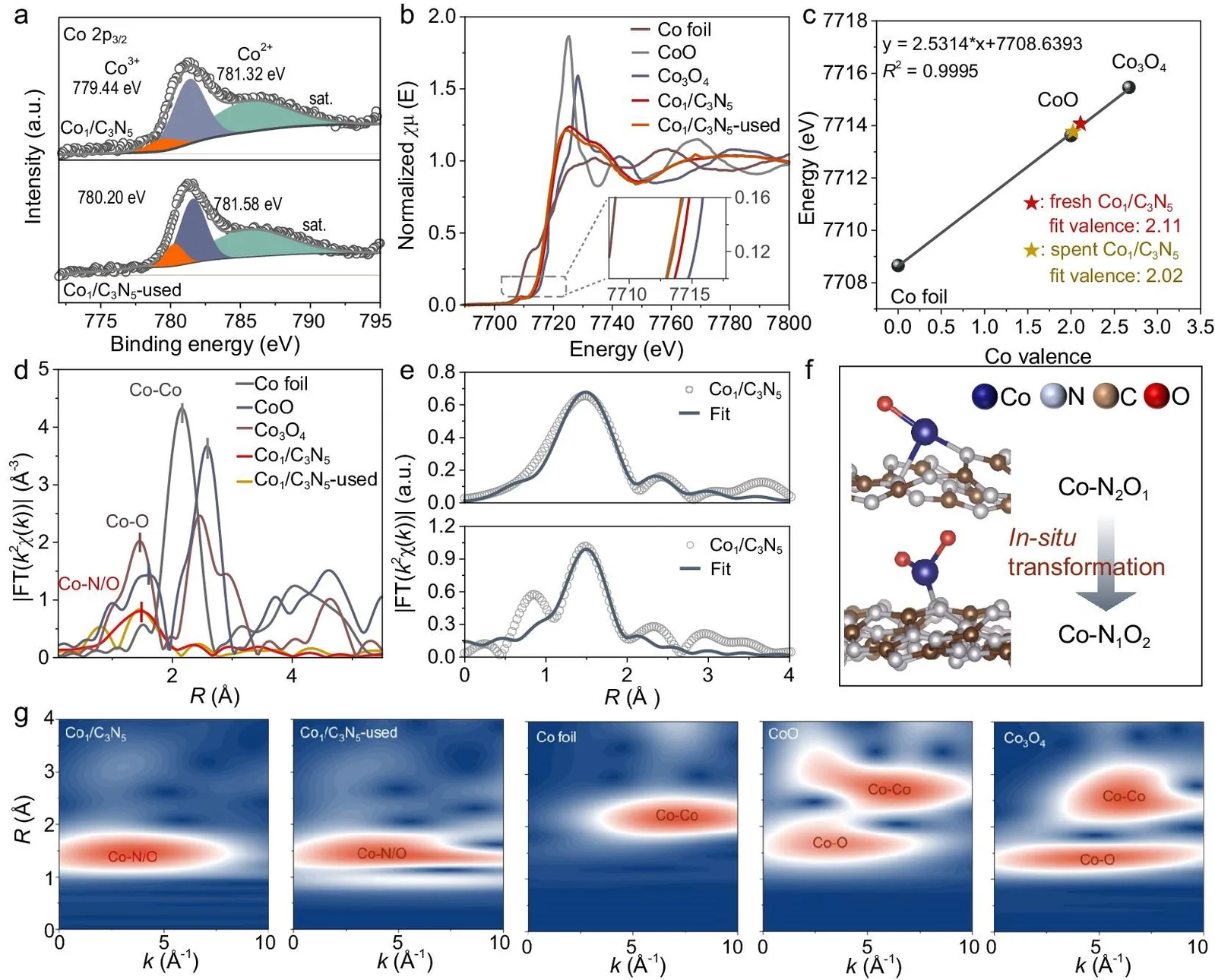
(a) Co 2p high-resolution XPS showing mixed Co^2+^/Co^3+^ features (surface-sensitive). (b and c) Co K-edge XANES and edge-position analysis indicating an average Co valence slightly above +2 (bulk-sensitive). (d) FT-EXAFS without Co–Co signature. (e) EXAFS fits supporting a Co–N_2_O_1_ environment in fresh samples and an O-richer first shell in spent samples. (f) Corresponding first-shell structural motifs. (g) Wavelet transform EXAFS (WT-EXAFS) corroborating O participation. Note: XPS (surface) vs. XANES/EXAFS (bulk) sensitivity accounts for apparent differences in oxidation-state descriptors.

The Co 2p XPS spectra (Fig. 2a and Fig. S7) reveal characteristic signatures of mixed-valent cobalt species, comprising both Co^2+^ and Co^3+^ oxidation states. To gain deeper insight into the electronic structure and coordination environment of Co centers, X-ray absorption spectroscopy was performed. The Co K-edge X-ray absorption near-edge structure (XANES) spectrum (Fig. 2b) of Co_1_/C_3_N_5_ displays an absorption edge positioned between those of CoO and metallic Co foil, corresponding to a mean cobalt oxidation state of approximately +2.11 (as derived from linear combination fitting) (Fig. 2c). The observed deviation between this result and the Co^2+^/Co^3+^ mixed valence state depicted in Fig. 2a can be attributed to the influence of Co atoms’ asymmetric coordination structure on the absorption edge position.

Fourier-transformed extended X-ray absorption fine structure (FT-EXAFS) spectra (Fig. 2d) exhibit a predominant coordination peak at ∼1.5 Å (uncorrected for phase shift), assignable to Co–N/O scattering paths. Importantly, no Co–Co coordination peak (∼2.2 Å) is observed, confirming the absence of cobalt clusters or metallic aggregates. Quantitative EXAFS fitting (Fig. 2e, Figs S8 and S9, and Table S1) indicates that the optimal model comprised a mixed coordination sphere with both nitrogen and oxygen ligands. Specifically, coordination numbers of ∼2.3 for N and ∼1.4 for O were determined at bond distances of 1.976 and 2.043 Å, respectively, implying a probable Co–N_2_O_1_ configuration. The proposed local coordination structure of the Co center is schematically illustrated in Fig. 2f. Moreover, wavelet transform (WT) EXAFS analysis (Fig. 2g) displays a maximum intensity shift toward lower R-space values relative to Co–N and Co–Co references, reinforcing the involvement of Co–O coordination and substantiating the incorporation of oxygen atoms within the coordination sphere [33,34].

Under PMS activation, ex-/post-reaction XANES/EXAFS/WT (Fig. 2b–g) indicate a shift toward an O-richer first shell, consistent with a Co–N_1_O_2_ configuration for the spent catalyst. We therefore refer to a reaction-induced coordination evolution from Co–N_2_O_1_ (fresh) to Co–N_1_O_2_ (spent), inferred from pre/post spectroscopies rather than direct operando proof [35]. This evolution—corroborated by our DFT structural models (Section DFT calculations)—serves as the structural basis for the spin-selection-favored PMS activation discussed below.

### PMS activation by Co_1_/C_3_N_5_ for degradation of ECs

The catalytic efficacy of Co_1_/C_3_N_5_ in activating PMS was systematically evaluated through the degradation of oxytetracycline (OTC) as a model EC. Given the dual necessity of maximizing degradation efficiency while minimizing metal loading for environmental and economic viability, catalysts with varied cobalt contents were screened. As shown in Fig. S10a and b, Co_1_/C_3_N_5_-2 exhibited an optimal balance between degradation rate, reaction kinetics, and metal utilization, and was therefore selected as the benchmark catalyst for subsequent studies.

**Figure 3.**
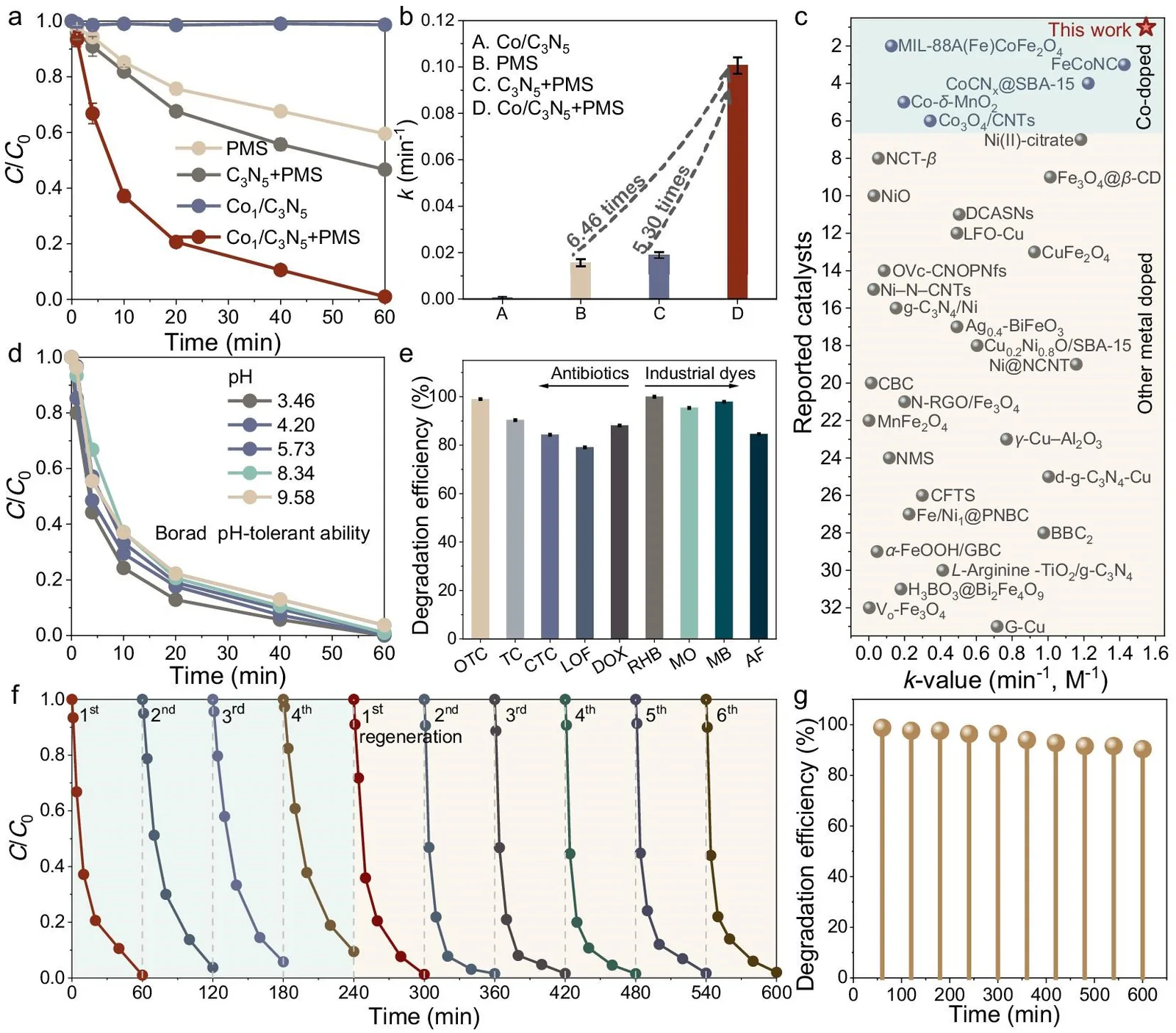
(a) Degradation profiles of OTC over time. (b) Corresponding OTC degradation rates. (c) Comparison of apparent rate constants (*k*) with previously reported catalysts. (d) Effect of initial pH on OTC degradation in the Co_1_/C_3_N_5_-activated PMS system. (e) Removal efficiencies of various organic pollutants using Co_1_/C_3_N_5_ for PMS activation. (f) Recycling performance of Co_1_/C_3_N_5_ for OTC degradation through successive reaction cycles. (g) The OTC removal efficiencies of simulated emergency wastewater by Co_1_/C_3_N_5_.

Control experiments demonstrated negligible OTC adsorption by Co_1_/C_3_N_5_ alone (1.29% removal), underscoring that degradation primarily stems from catalytic activation of PMS rather than physisorption (Fig. 3a). In the presence of PMS, pristine C_3_N_5_ achieved 53.34% OTC removal within 90 min, whereas Co_1_/C_3_N_5_ attained an outstanding removal efficiency of 98.99% under identical conditions, unequivocally demonstrating the catalytic role of Co centers in PMS activation. Furthermore, the degradation efficiency of Co_1_/C_3_N_5_ was significantly higher than that of Co^2+^ (42.2%), indicating that anchored Co atoms possess stronger PMS activation capabilities than free Co^2+^ (Fig. S11). Kinetic analysis (Fig. 3b) revealed that the pseudo-first-order rate constant (*k*) for Co_1_/C_3_N_5_ was 0.10 min^−1^—5.3-fold higher than that of C_3_N_5_ alone. Furthermore, Co_1_/C_3_N_5_ exhibited an excellent turnover frequency (*k*) of 1.548 min^−1^ M^−1^, surpassing many state-of-the-art catalysts reported in literature (Fig. 3c and Table S2). This *k* value was determined based on parameters including catalyst concentration, PMS concentration, pollutant concentration, and reaction rate, using the normalized calculation formula provided in Table S2, thereby ensuring a scientifically sound comparison of performance across different research systems. These results provide compelling evidence that the Co-doped C_3_N_5_ catalyst effectively promotes the generation of highly oxidized species and demonstrates outstanding performance in activating PMS (Fig. S12).

The robustness of Co_1_/C_3_N_5_ under diverse water matrices was probed. The catalyst maintained high OTC degradation efficiencies in tap water (92.42%), river water (90.47%), and rain water (89.33%) (Fig. S13). Tap water and river water promote OTC removal during the early phase of the reaction, attributed to the presence of Cl^−^ in the water, which can react with PMS to generate ROS. Moreover, the system exhibited high resilience against common anions (HCO_3_^−^, CO_3_^2−^, Cl^−^, and NO_3_^−^) (Figs S14 and S15), and sustained excellent performance over a broad pH range (3.0–10.0), with negligible activity loss (Fig. 3d). Cl^−^ exhibits a slight promoting effect on OTC degradation. According to the reported literature [36], this is because Cl^−^ reacts with PMS to generate reactive chlorine species (for example, Cl_2_, ClO^−^), which in turn attack PMS, inducing the production of more ^1^O_2_. The specific mechanism is shown in Equations (1–4) [36,37]. These features highlight its adaptability to complex water chemistries typically encountered in practical applications:


(1) \begin{eqnarray*}
{\mathrm{C}}{{\mathrm{l}}}^ - + \cdot {\mathrm{S}}{{\mathrm{O}}}_4^ {\ \ -} \to \cdot {\mathrm{Cl}} + {\mathrm{S}}{{\mathrm{O}}}_4{^2}^ - ,
\end{eqnarray*}

(2) \begin{eqnarray*}
\cdot {\mathrm{Cl}} + \cdot {\mathrm{Cl}} \to {\mathrm{C}}{{\mathrm{l}}}_2,
\end{eqnarray*}

(3) \begin{eqnarray*}
{\mathrm{C}}{{\mathrm{l}}}^ {\ \ -} + {\mathrm{HS}}{{\mathrm{O}}}_5^ - \to {\mathrm{S}}{{\mathrm{O}}}_4{^2}^ - + {\mathrm{HClO}},
\end{eqnarray*}

(4) \begin{eqnarray*}
&&{\mathrm{C}}{{\mathrm{l}}}_2 + {\mathrm{HS}}{{\mathrm{O}}}_5^ {\ \ -} + {{\mathrm{H}}}_2{\mathrm{O}} \to {\mathrm{S}}{{\mathrm{O}}}_4{^2}^ - + 2{\mathrm{C}}{{\mathrm{l}}}^ - \\
&&\quad\quad\quad +\, 3{{\mathrm{H}}}^ + { + }^1{{\mathrm{O}}}_2.
\end{eqnarray*}

The versatility of the Co_1_/C_3_N_5_/PMS system was further evaluated using a range of structurally distinct organic pollutants. As shown in Fig. 3e and Fig. S16, representative dyes, including RhB (100.00%, 0.069 min^−1^), MO (95.39%, 0.110 min^−1^), MB (97.92%, 0.093 min^−1^), and AF (84.52%, 0.021 min^−1^), were efficiently removed within 60 min. Similarly, typical antibiotics, including TC (90.36%, 0.071 min^−1^), CTC (84.31%, 0.050 min^−1^), LOF (79.13%, 0.039 min^−1^), and DOX (88.14%, 0.091 min^−1^), also underwent rapid degradation. These results demonstrate the broad-spectrum oxidation capability of Co_1_/C_3_N_5_ toward pollutants with diverse molecular structures, highlighting its potential for the treatment of complex organic wastewater.

Catalyst durability was evaluated through four consecutive degradation cycles (Fig. 3f). Co_1_/C_3_N_5_ maintained 98.44% OTC removal efficiency after four cycles, indicating excellent stability. The slight decrease in activity was attributed mainly to partial blockage of active sites by adsorbed intermediates. Thermal regeneration (300°C, 3 h, static air) effectively restored the catalytic performance and even led to a faster degradation rate after regeneration. To clarify whether this improvement originated from structural changes of the active sites, Co K-edge XAFS characterization was further performed on the regenerated catalyst (Fig. S17 and Table S4). The fitting results show that the coordination environment of Co_1_/C_3_N_5_-regeneration is highly consistent with that of Co_1_/C_3_N_5_-used, and both are best assigned to a Co–N_1_O_2_ configuration. This observation demonstrates that thermal regeneration does not induce further reconstruction of the Co coordination structure, but instead restores catalytic activity mainly by removing surface-adsorbed intermediates and re-exposing the Co active sites. These results further confirm that the reaction-induced transformation from Co–N_2_O_1_ to Co–N_1_O_2_ is stable and irreversible under the present catalytic conditions.

The continuous-flow treatment process achieved a 90.36% OTC removal efficiency within a 600 min operational period, demonstrating outstanding catalytic performance (Fig. 3g and Fig. S18). Post-reaction structural integrity of Co_1_/C_3_N_5_ was confirmed by XRD and XPS analyses. XRD patterns (Fig. S19a) reveal that the crystalline C_3_N_5_ phase is well retained after use and regeneration. XPS spectra (Fig. S19b–f) show that the surface chemical states remain largely stable after reaction, although a modest increase in the Co^2+^ fraction is observed (Fig. S20), suggesting slight electronic modulation during catalytic cycling. In addition, the binding energy of the N–C=N component in the used Co_1_/C_3_N_5_ sample shifts to a higher value (Fig. S19d). This change is attributed to the evolution of the Co coordination environment during reaction, in which partial coordination with more electronegative O species decreases the local electron density of the surrounding framework. Meanwhile, adsorption of ROS on the catalyst surface may also contribute to the increase in surface oxygen-related species, thereby further affecting the electronic environment of the N–C=N moiety.

To probe the coordination environment of Co center’s post-reaction, X-ray absorption spectroscopy was conducted [38,39]. XANES spectra (Fig. 2b and c) revealed that the Co valence state remained between 0 and +2 after use. FT-EXAFS analysis (Fig. 2d) indicated a prominent Co–N/O coordination peak at ≈1.47 Å, distinct from metallic Co–Co interactions. Quantitative EXAFS fitting (Fig. S9 and Table S3) determined that used Co_1_/C_3_N_5_ adopts a Co–N_1_O_2_ coordination motif, with bond lengths of 2.008 Å (Co–N) and 2.191 Å (Co–O), differing from the initial Co–N_2_O_1_ structure (Fig. 2f). WT EXAFS spectra (Fig. 2g) further corroborated this structural adaptation. The configuration of the catalyst after regeneration is consistent with this coordination geometry, highlighting the irreversible and stable structural rearrangement of the active site under reaction-induced conditions.

The regenerated catalyst retains a coordination configuration consistent with that of the reaction-induced active site, indicating that the structural rearrangement is irreversible yet stable under catalytic conditions. Moreover, the FT-EXAFS profiles (Figs 2d and S17b) and WT-EXAFS spectra (Fig. 2g and Fig. S17g) of the fresh, used, and regenerated samples show no detectable Co–Co coordination signal after PMS activation, effectively excluding metal aggregation during the reaction. Together with the XRD and XPS results, these findings demonstrate that Co_1_/C_3_N_5_ can maintain atomically dispersed Co sites even under harsh redox conditions, evidencing the exceptional structural stability of its coordination environment.

These findings reveal that Co_1_/C_3_N_5_ undergoes adaptive coordination restructuring during PMS activation, dynamically shifting from Co–N_2_O_1_ to Co–N_1_O_2_ environments to facilitate sustained PMS activation. This *in situ* structural flexibility underpins the exceptional stability and catalytic robustness of Co_1_/C_3_N_5_ for EC degradation.

### Condition optimization for PMS activation by Co_1_/C_3_N_5_

To assess the operational feasibility and scalability of the Co_1_/C_3_N_5_/PMS system for pollutant removal, systematic condition-variation experiments were performed. The influence of PMS concentration, catalyst dosage, and pollutant loading on OTC degradation was thoroughly investigated using the optimized Co_1_/C_3_N_5_ catalyst. As depicted in Fig. S21a, increasing PMS concentration (0.05–0.4 mg mL^−1^) progressively enhanced OTC degradation efficiency. However, the rate of improvement plateaued as PMS dosage rose from 0.2 to 0.4 mg mL^−1^, likely due to saturation of available catalytic active sites. This suggests that beyond a threshold PMS concentration, excess oxidant does not proportionally contribute to ROS generation, possibly due to self-quenching phenomena or active-site limitations.

Similarly, increasing catalyst dosage (Fig. S21b) positively influenced OTC removal efficiency. A noticeable deceleration in performance gain was observed when catalyst loading exceeded 1.2 mg mL^−1^, indicating that this dosage is sufficient to fully activate 0.2 mg mL^−1^ PMS under the studied conditions. Excess catalyst beyond this optimal loading confers marginal benefit, likely due to either particle aggregation reducing accessible surface area or diminishing returns in ROS generation relative to oxidant availability. Furthermore, OTC concentration exerted a strong impact on degradation kinetics. As shown in Fig. S21c, lower initial OTC concentrations yielded higher removal efficiencies under fixed catalyst and PMS dosages. This inverse relationship highlights that under constant catalytic conditions, pollutant degradation proceeds more rapidly at lower substrate concentrations due to reduced competition for reactive species and diminished mass transfer limitations.

Collectively, these optimization experiments delineate the following practical operating conditions for maximal catalytic performance: 0.2 mg mL^−1^ PMS, 1.2 mg mL^−1^ Co_1_/C_3_N_5_ catalyst, and pollutant concentrations preferably below 20 mg L^−1^. These insights provide a rational foundation for translating laboratory efficacy toward real-world water treatment applications, balancing catalytic efficiency with economic and operational considerations.

### Selective generation of reactive species during PMS activation by Co_1_/C_3_N_5_

To elucidate the mechanistic pathways underpinning PMS activation by Co_1_/C_3_N_5_ and C_3_N_5_ catalysts, a combination of quenching experiments and electron paramagnetic resonance (EPR) spectroscopy was employed to systematically identify both radical and non-radical reactive species in the reaction system.

**Figure 4.**
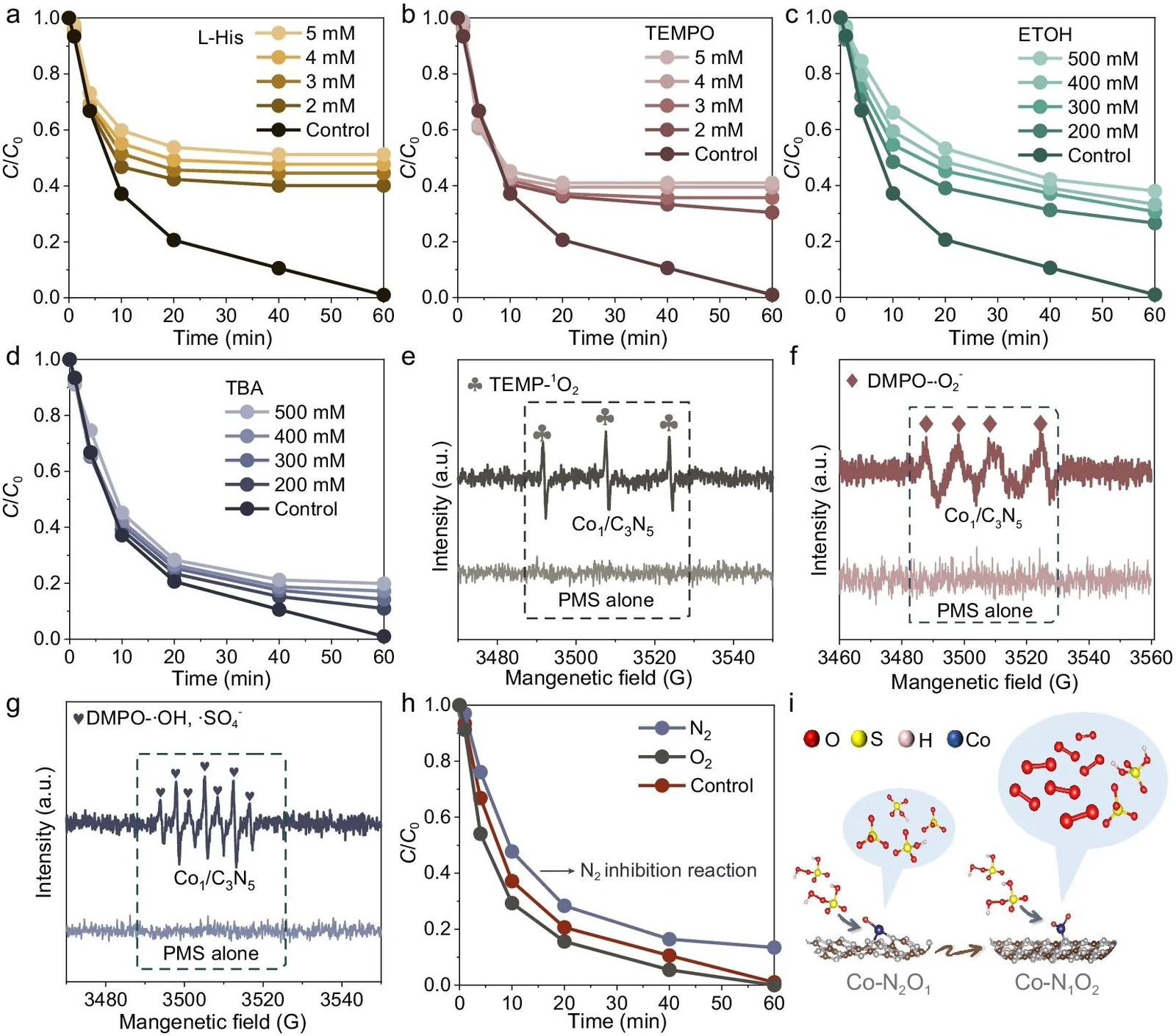
(a–d) Effects of various scavenger dosages on OTC degradation in the Co_1_/C_3_N_5_-activated PMS system: (a) *L*-histidine (*L*-His), (b) 2,2,6,6-tetramethylpiperidine-1-oxyl (TEMPO), (c) ethanol (EtOH), and (d) *tert*-butanol (TBA). (e–g) EPR spectra of (e) TEMP–^1^O_2_, (f) DMPO–·O_2_^−^, and (g) DMPO–·OH/·SO_4_^−^ in PMS-only and Co_1_/C_3_N_5_-coupled PMS systems, identifying ROS. (h) Degradation profiles of OTC under various atmospheric conditions in the Co_1_/C_3_N_5_/PMS system, highlighting the role of dissolved gases. (i) Proposed reaction mechanism illustrating the pathways of PMS activation and OTC degradation mediated by Co_1_/C_3_N_5_.

Four selective scavengers were utilized to probe distinct reactive intermediates: *L*-histidine (*L*-His) for singlet oxygen (^1^O_2_), 1-*λ*¹-oxidanyl-2,2,6,6-tetramethylpiperidin-4-ol (TEMPO) for superoxide radicals (·O_2_^−^), ethanol (EtOH) for sulfate radicals (·SO_4_^−^), and tert-butanol (TBA) for hydroxyl radicals (·OH). In the Co_1_/C_3_N_5_/PMS system, the addition of all four scavengers led to discernible suppression of OTC degradation (Fig. 4a–d). Specifically, increasing the concentrations of *L*-His and TEMPO from 2 to 5 mM reduced OTC removal from 59.86% and 69.57% to 48.75% and 58.97%, respectively. Similarly, raising EtOH and TBA concentrations from 200 to 500 mM decreased OTC removal from 73.44% and 98.02% to 61.97% and 80.12%, respectively (Fig. 4c and d). These findings confirm the co-existence and contribution of four distinct reactive species to pollutant degradation. Notably, the relative importance of these species in the Co_1_/C_3_N_5_/PMS system follows the order: ^1^O_2_ (37.53%) > ·O_2_^−^ (26.00%) > ·SO_4_^−^ (24.74%) > ·OH (11.91%) (Fig. S22). In contrast, for the C_3_N_5_/PMS system (Fig. S23), only EtOH and TBA significantly inhibited OTC removal, indicating that ⋅OH and ·SO_4_^−^ were the predominant species involved, whereas ^1^O_2_ and ·O_2_^−^ were negligible. Furthermore, dimethyl sulfoxide (DMSO) was employed as a probe for high-valent cobalt–oxo species. The observed inhibition of OTC degradation after DMSO addition in the Co_1_/C_3_N_5_/PMS system (Fig. S24a) suggests the possible involvement of Co^IV^=O-like species in pollutant oxidation. To further probe this possibility, *in situ* Raman spectroscopy was performed under reaction conditions. As shown in Fig. S24b, a new Raman band gradually emerged at around 570 cm^−1^ with increasing reaction time. According to previous studies, this feature may be associated with high-valent cobalt–oxo species formed during PMS activation [40,41]. Although this assignment should be interpreted with appropriate caution, the Raman results, together with the quenching experiments, support the possible participation of Co^IV^=O-like intermediates in the catalytic oxidation process.

EPR spectroscopy provided direct evidence corroborating these observations. As shown in Fig. 4e–g, characteristic signals corresponding to TEMP-^1^O_2_, DMPO-·O_2_^−^, DMPO-·OH, and DMPO-·SO_4_^−^ adducts were all discernible in the Co_1_/C_3_N_5_/PMS system, substantiating the formation of both radical (·OH, ·SO_4_^−^, and ·O_2_^−^) and non-radical (^1^O_2_) species [42]. This multi-channel activation capacity distinguishes Co_1_/C_3_N_5_ as a highly versatile PMS activator. To further elucidate the origin of reactive species, particularly oxygen-containing intermediates, the role of dissolved oxygen (DO) was systematically investigated. As shown in Fig. 4h, removal of DO by N_2_ purging significantly suppressed OTC degradation, whereas O_2_ enrichment further enhanced the catalytic performance, indicating that DO plays an important role in the generation of ROS. Combined with the quenching and EPR results, these observations suggest that DO is involved in the formation of ^1^O_2_ and ·O_2_^−^ species. To clarify whether DO directly affects oxidant consumption, PMS decomposition under different atmospheric conditions was also examined. The results show that DO has only a minimal influence on PMS consumption in the Co_1_/C_3_N_5_/PMS system (Fig. S25), indicating that its primary contribution lies in promoting ROS generation rather than accelerating PMS decomposition.

Based on these collective insights, a comprehensive mechanistic scheme is proposed (Fig. 4i). Upon PMS activation by atomically dispersed Co centers, both homolytic and heterolytic cleavage of PMS occurs, generating ·SO_4_^−^·and ·OH via conventional pathways. Simultaneously, Co-induced PMS activation promotes oxygen transfer processes involving DO, facilitating the generation of ^1^O_2_ and ·O_2_^−^ through sequential electron-transfer and energy-transfer routes. The unique Co–N/O coordination environment in Co_1_/C_3_N_5_ serves as a bifunctional active site that mediates PMS decomposition and oxygen activation synergistically. This structural modulation governs both the type and abundance of reactive species, thereby dictating the degradation pathway of OTC and potentially other organic pollutants. These mechanistic insights highlight how atomic Co incorporation transforms the reactivity landscape, expanding beyond traditional radical pathways to enable non-radical processes, ultimately contributing to the catalyst’s superior oxidative performance and versatility in environmental applications.

### DFT calculations elucidating the PMS activation mechanism at Co–N_2_O_1_ atomic sites

**Figure 5.**
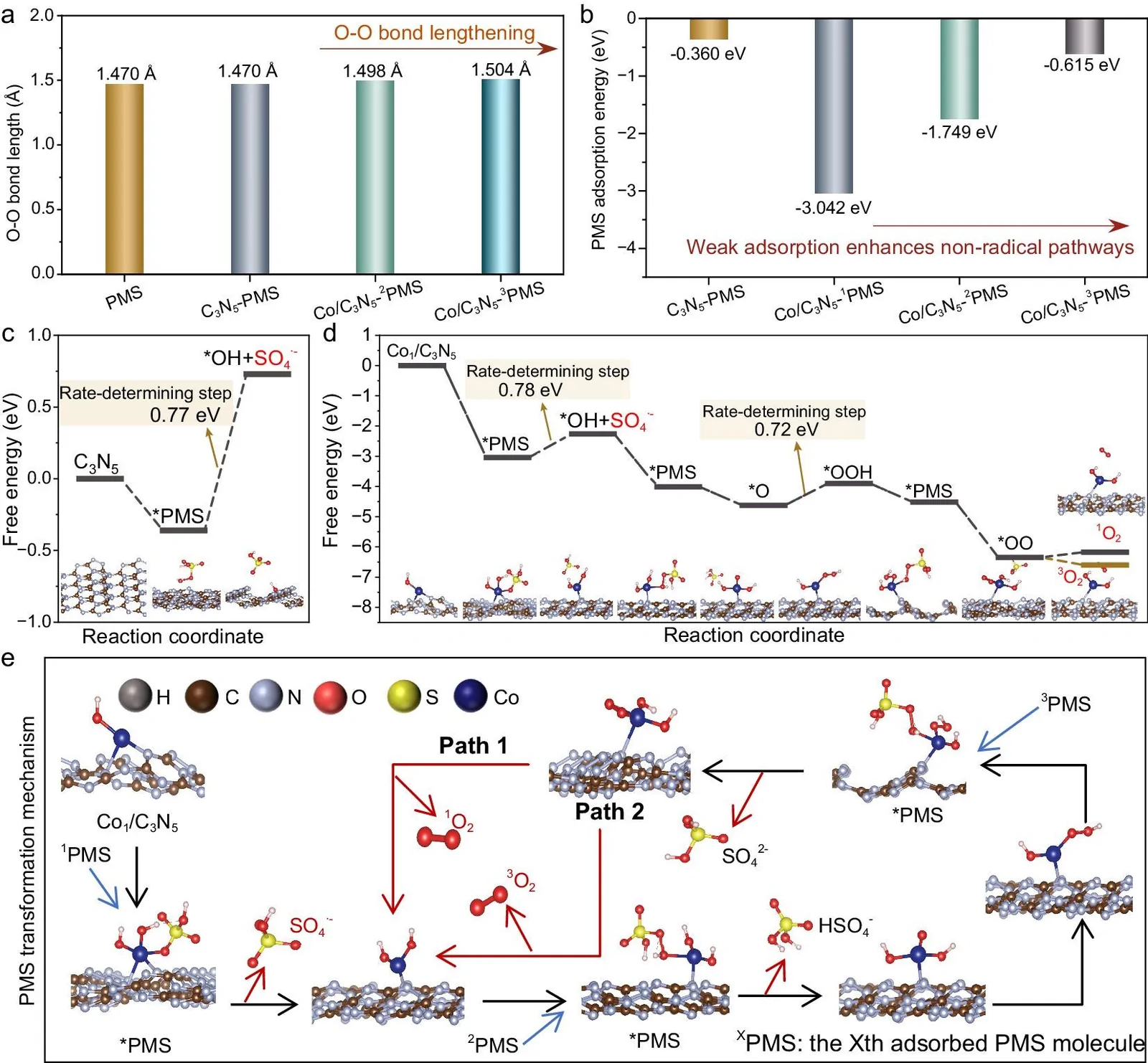
(a) O–O bond lengths of PMS in its free state and upon adsorption on C_3_N_5_ and Co_1_/C_3_N_5_. (b) Calculated adsorption energies of PMS on C_3_N_5_ and Co_1_/C_3_N_5_. (c and d) Reaction pathways and corresponding free energy profiles for PMS activation, illustrating the generation of ^1^O_2_, ^3^O_2_, and ·SO_4_^−^ species. Insets depict key intermediates adsorbed at C_3_N_5_ and Co_1_/C_3_N_5_ active sites. (e) Schematic illustration of PMS adsorption and decomposition on Co_1_/C_3_N_5_, leading to the production of ^1^O_2_, ^3^O_2_, and ·SO_4_^−^ reactive species.

To unravel the atomic-level mechanisms governing PMS activation on Co_1_/C_3_N_5_, DFT calculations were conducted using simplified structural models of pristine C_3_N_5_ and Co-anchored C_3_N_5_. These theoretical investigations aimed to dissect the structural stability, adsorption behavior, bond activation, and reactive intermediate generation induced by atomically dispersed Co sites. The *ab initio* molecular dynamics simulations were employed to reflect atomic structures and chemical environments from an electronic structure perspective. The result shows that structures of Co–N_2_O_1_ and Co–N_1_O_2_ exhibit stabilized energy profiles within 0–10 000 fs, demonstrating the structural stability and reliability (Fig. S26). As illustrated in Fig. 5a, the optimized configurations reveal that PMS adsorption on Co_1_/C_3_N_5_ induces a pronounced elongation of the O–O bond (L_O–O_) relative to both free PMS (1.470 Å) and PMS adsorbed on C_3_N_5_ (1.470 Å). This bond elongation signifies efficient PMS activation facilitated by Co–N_2_O_1_ sites, owing to enhanced electron density transfer and bond polarization. Consistently, calculated adsorption energies indicate that PMS exhibits substantially stronger affinity for Co_1_/C_3_N_5_ than for pristine C_3_N_5_.

The calculated binding strength follows the order Co_1_/C_3_N_5_−^1^PMS > Co_1_/C_3_N_5_−^2^PMS > Co_1_/C_3_N_5_−^3^PMS > C_3_N_5_−PMS (Fig. 5b), revealing a gradual attenuation of PMS adsorption upon sequential occupation of the Co site. At the initial stage, the relatively strong interaction between PMS and the Co center facilitates O–O bond activation and favors homolytic cleavage, thereby promoting the radical pathway, particularly the formation of ·SO_4_^−^. As PMS adsorption progressively weakens, the O–O bond cleavage tends to shift toward a heterolytic mode, which is more favorable for the generation of HSO_4_^−^ and ^1^O_2_. This adsorption-energy-dependent evolution of the reaction pathway is consistent with the experimentally observed ROS distribution and provides a mechanistic basis for the superior catalytic performance of Co_1_/C_3_N_5_.

To deepen mechanistic understanding, the free energy changes associated with the formation of key reactive intermediates—^1^O_2_, ·O_2_^−^, ·SO_4_^−^, and ·OH—were calculated for both Co_1_/C_3_N_5_ and C_3_N_5_ systems (Fig. 5c and d). The formation of ·O_2_^−^ can occur through electron acquisition by O_2_, whose stable ground state is triplet oxygen (^3^O_2_). Therefore, the role of ·O_2_^−^ in Co_1_/C_3_N_5_/PMS system can be elucidated by investigating ^3^O_2_. The results reveal that Co_1_/C_3_N_5_ thermodynamically favors the generation of ^1^O_2_ and ·O_2_^−^, whereas ·SO_4_^−^, and ·OH formation pathways are comparatively less favorable. This theoretical prediction dovetails with experimental findings from scavenger tests, EPR analyses, and DO-purging experiments, collectively confirming that atomic Co incorporation steers PMS activation toward alternative, energetically favorable oxygen transfer pathways.

Based on these combined computational and experimental insights, a comprehensive mechanistic scheme for PMS activation and subsequent pollutant degradation is proposed (Fig. 5e). The key reaction steps are summarized as follows [43,44]:


(5) \begin{eqnarray*}
{\mathrm{C}}{{\mathrm{o}}}^{2 + } + {\mathrm{HS}}{{\mathrm{O}}}_5^ {\ \ -} \to {\mathrm{C}}{{\mathrm{o}}}^{3 + } + {\mathrm{O}}{{\mathrm{H}}}^ - + \cdot {\mathrm{S}}{{\mathrm{O}}}_4^ {\ \ -} ,
\end{eqnarray*}

(6) \begin{eqnarray*}
{\mathrm{C}}{{\mathrm{o}}}^{2 + } + {\mathrm{HS}}{{\mathrm{O}}}_5^ {\ \ -} \to {\mathrm{C}}{{\mathrm{o}}}^{3 + } + \cdot {\mathrm{OH}} + {\mathrm{S}}{{\mathrm{O}}}_4^{\ \ 2 - },
\end{eqnarray*}

(7) \begin{eqnarray*}
\cdot {\mathrm{S}}{{\mathrm{O}}}_4^ {\ \ -} + {{\mathrm{H}}}_2{\mathrm{O}} \to {\mathrm{S}}{{\mathrm{O}}}_4^{\ \ 2 - } + \cdot {\mathrm{OH}} + {{\mathrm{H}}}^ + ,
\end{eqnarray*}

(8) \begin{eqnarray*}
{\mathrm{C}}{{\mathrm{o}}}^{3 + } + {{\mathrm{e}}}^ - \to {\mathrm{C}}{{\mathrm{o}}}^{2 + },
\end{eqnarray*}

(9) \begin{eqnarray*}
{\mathrm{C}}{{\mathrm{o}}}^{3 + } + {\mathrm{HS}}{{\mathrm{O}}}_5^ {\ \ -} \to {\mathrm{C}}{{\mathrm{o}}}^{2 + } + \cdot {\mathrm{S}}{{\mathrm{O}}}_5^ {\ \ -} + {{\mathrm{H}}}^ + ,
\end{eqnarray*}

(10) \begin{eqnarray*}
{\mathrm{HS}}{{\mathrm{O}}}_5^ {\ \ -} \to {\mathrm{S}}{{\mathrm{O}}}_5^{\ \ 2 - } + {{\mathrm{H}}}^ + ,
\end{eqnarray*}

(11) \begin{eqnarray*}
{\mathrm{HS}}{{\mathrm{O}}}_5^ {\ \ -} + {\mathrm{S}}{{\mathrm{O}}}_5^{\ \ 2 - } \to {\mathrm{HS}}{{\mathrm{O}}}_4^ {\ \ -} + {\mathrm{S}}{{\mathrm{O}}}_4^{\ \ 2 - }{ + }^1{{\mathrm{O}}}_2,
\end{eqnarray*}

(12) \begin{eqnarray*}
\cdot {\mathrm{S}}{{\mathrm{O}}}_5^ {\ \ -} + \cdot {\mathrm{S}}{{\mathrm{O}}}_5^ {\ \ -} \to {\mathrm{S}}{{\mathrm{O}}}_4^{\ \ 2 - } + {\mathrm{S}}{{\mathrm{O}}}_4^{\ \ 2 - }{ + }^1{{\mathrm{O}}}_2,
\end{eqnarray*}

(13) \begin{eqnarray*}
\cdot {\mathrm{S}}{{\mathrm{O}}}_5^ {\ \ -} + \cdot {\mathrm{S}}{{\mathrm{O}}}_5^ {\ \ -} \to {{\mathrm{S}}}_2{{\mathrm{O}}}_8{^2}^ - { + }^1{{\mathrm{O}}}_2,
\end{eqnarray*}

(14) \begin{eqnarray*}
\cdot {\mathrm{S}}{{\mathrm{O}}}_5^ {\ \ -} + \cdot {\mathrm{S}}{{\mathrm{O}}}_5^ {\ \ -} + {{\mathrm{H}}}_2{\mathrm{O}} \to 2\,{\mathrm{HS}}{{\mathrm{O}}}_4^ {\ \ -} + {1.5}^1{{\mathrm{O}}}_2,
\end{eqnarray*}

(15) \begin{eqnarray*}
&&{\mathrm{C}}{{\mathrm{o}}}^{2 + } + {\mathrm{HS}}{{\mathrm{O}}}_5^ {\ \ -} + {{\mathrm{H}}}_2{\mathrm{O}} \to {\mathrm{C}}{{\mathrm{o}}}^{3 + } + \cdot {{\mathrm{O}}}_2^ {\ \ -}\\
&&\quad\quad\quad +\ {\mathrm{HS}}{{\mathrm{O}}}_4^ {\ \ -} + 2{\rm H}^+,
\end{eqnarray*}

(16) \begin{eqnarray*}
{\mathrm{S}}{{\mathrm{O}}}_5^{\ \ 2 - } + {{\mathrm{H}}}_2{\mathrm{O}} \to \cdot {{\mathrm{O}}}_2^ {\ \ -} + {\mathrm{S}}{{\mathrm{O}}}_4^{\ \ 2 - } + {{\mathrm{H}}}^ + .
\end{eqnarray*}

Notably, DFT calculations indicate that the local coordination environment of Co evolves from Co–N_2_O_1_ to Co–N_1_O_2_ during PMS activation. The initial Co–N_2_O_1_ site strongly adsorbs the first PMS molecule with an adsorption energy of 3.042 eV, followed by dissociation at the Co center, which induces reconstruction of the local coordination structure into Co–N_1_O_2_. Subsequently, the second and third PMS molecules adsorb on the Co–N_1_O_2_ surface with adsorption energies of 1.749 and 0.615 eV, respectively, and their O–O bonds are elongated to 1.498 and 1.504 Å, indicating effective activation. These results suggest that the evolved Co–N_1_O_2_ motif provides a more favorable local environment for subsequent PMS activation and oxygen-related intermediate formation. Considering the DFT results together with the observed ^1^O_2_-dominant oxidation behavior, we propose that this coordination evolution is closely associated with the spin-selection-favored PMS activation pathway. In this sense, the Co–N_2_O_1_ → Co–N_1_O_2_ transformation likely contributes to a catalytic microenvironment that is more favorable for *OO intermediate formation and subsequent ^1^O_2_ generation. This interpretation is further supported by EXAFS analysis (Fig. 2e and f), which confirms the *in situ* evolution of the Co coordination structure under reaction conditions.

Collectively, these findings establish that atomically dispersed Co sites not only enhance PMS adsorption and cleavage but also selectively promote oxygen transfer routes leading to ^1^O_2_ and ·O_2_^−^ generation, while simultaneously enabling structural adaptability to maintain catalytic activity. This dual functionality of Co sites—chemical reactivity and structural plasticity—underpins the superior catalytic performance of Co_1_/C_3_N_5_ in PMS-based AOPs.

### Spin-polarized DFT elucidation of spin-state governed ^1^O_2_ generation pathways

**Figure 6.**
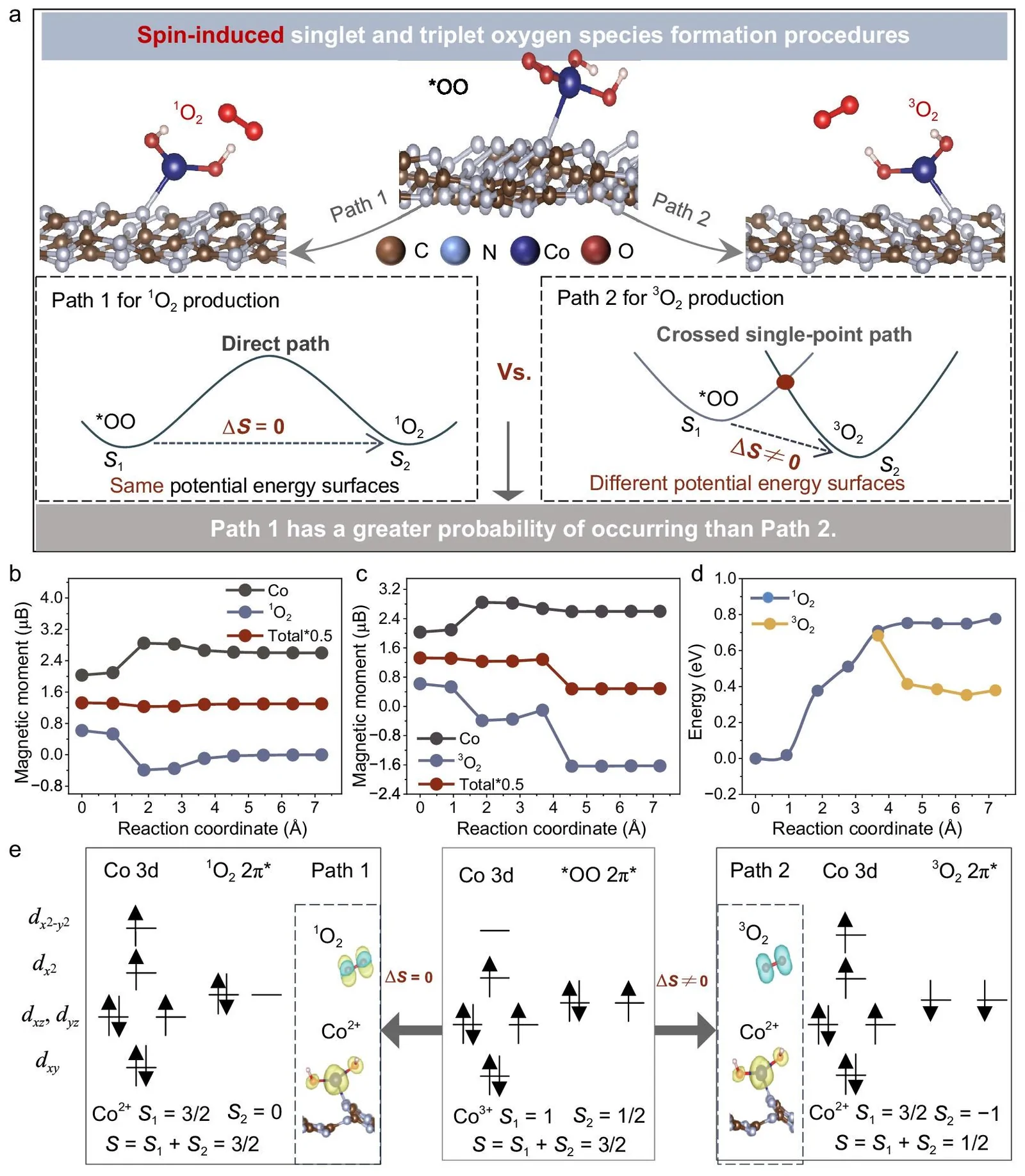
(a) Schematic illustration of the reaction pathways and corresponding potential energy surfaces for *OO transformation into ^1^O_2_ and ^3^O_2_. (b and c) Evolution of magnetic moments along path 1 and path 2. (d) Calculated MEPs for the formation of ^1^O_2_ and ^3^O_2_. (e) Schematic representations of the spin configurations of Co 3d orbitals, *OO intermediate, and the resulting ^1^O_2_ and ^3^O_2_ species. Insets show charge density difference maps, where light and dark regions denote electron accumulation and depletion, respectively.

To unravel the influence of Co spin states and spin-selection rules on the transformation of surface-bound *OO intermediates into singlet oxygen (^1^O_2_) and triplet oxygen (^3^O_2_), spin-polarized DFT calculations were performed [45,46]. Specifically, detailed magnetic moment analyses were conducted along two mechanistic trajectories (Path 1 and Path 2) as illustrated in Fig. 6a.

The computed magnetic moments indicate that along Path 1 (leading to ^1^O_2_ generation), the total magnetic moment of the Co_1_/C_3_N_5_/PMS system remains invariant (Fig. 6b), whereas along Path 2 (leading to ^3^O_2_), a marked change in total magnetic moment is observed (Fig. 6c). According to spin selection rules, transitions between states with differing total spin (Δ*S* ≠ 0) are quantum mechanically forbidden, while transitions conserving total spin (Δ*S* = 0) are symmetry-allowed and kinetically favored. Although spin-forbidden transitions can occur via spin–orbit coupling or thermal perturbations, they are generally less probable. Consequently, these findings suggest that *OO is intrinsically predisposed to convert into ^1^O_2_ via Path 1, whereas the formation of ^3^O_2_ via Path 2 is kinetically hindered due to spin mismatch.

Complementary analysis of the minimum energy paths (MEPs) further substantiates this hypothesis (Fig. 6d). Along Path 1, the *OO detachment from the Co site proceeds smoothly on a single potential energy surface, indicating a concerted and energetically facile route to ^1^O_2_. Conversely, the Path 2 trajectory involves an energetic discontinuity, indicative of an inter-surface crossing, which necessitates surmounting an additional energy barrier—thus rendering the generation of ^3^O_2_ both kinetically and thermodynamically less favorable.

The spin density configurations of the initial and final states (Fig. 6e) offer further mechanistic granularity. Remarkably, following *OO detachment, the spin state of Co transitions from a low-spin configuration (*S* = 1) to a high-spin state (*S* = 3/2), accompanied by an increase in the number of unpaired electrons at the Co center. This spin-state transition augments the electronic flexibility of the Co site, thereby facilitating electron transfer processes and enhancing subsequent PMS adsorption and activation [47]. A comprehensive mechanistic scheme based on spin theory is presented in Fig. 6a, delineating the superior efficacy of spin-selective *OO conversion in promoting ^1^O_2_ generation. These insights explicitly highlight the pivotal role of spin engineering strategies in tailoring catalytic PMS activation pathways, thereby offering a novel conceptual framework for the rational design of advanced oxygen transfer catalysts.

### Degradation pathway elucidation and ecotoxicological assessment

To comprehensively elucidate the degradation mechanism and evaluate the environmental safety of the Co_1_/C_3_N_5_-activated PMS system, intermediates generated during OTC degradation were identified using high-performance liquid chromatography-mass spectrometry (HPLC-MS). Based on the HPLC-MS results (Figs S27–S29), combined with ROS identification experiments and relevant literature, the degradation of OTC in the Co_1_/C_3_N_5_/PMS system is proposed to involve the synergistic action of ^1^O_2_, ·O_2_^−^, ·SO_4_^−^, and ·OH. Specifically, ^1^O_2_ and ·SO_4_^−^ are likely associated with oxidation at electron-rich sites in OTC, such as aromatic rings, C=C bonds, and amino-containing groups, which may induce dehydration, dehydroxylation, deamidation, and ring-opening reactions, leading to intermediates such as P4 (*m/z* = 447.14) and P11 (*m/z* = 279.16) [48–50]. In addition, ·O_2_^−^ may participate in direct oxidation of susceptible groups and may also contribute indirectly through conversion into ^1^O_2_, possibly relating to the formation of intermediates such as P12 (*m/z* = 204.12) [51]. Meanwhile, ·OH, as a highly reactive and nonselective oxidant, is likely involved in aromatic ring attack and in demethylation, hydroxylation, and deoxygenation processes, which may be associated with intermediates such as P1 (*m/z* = 470.19), P5 (*m/z* = 430.11), and P8 (*m/z* = 351.17) [52]. Through these sequential oxidation steps, OTC is proposed to undergo bond cleavage, ring opening, and progressive transformation into smaller oxygenated products.

To assess the ecological safety of the resultant degradation products, key toxicological indices—including acute toxicity, mutagenicity, bioaccumulation potential, and developmental toxicity—were systematically evaluated using the U.S. EPA Toxicity Estimation Software Tool (T.E.S.T.). As summarized in Fig. S30a, 68% of the identified intermediates exhibited diminished developmental toxicity, with individual reductions ranging from 1.20% to 39.76%. Moreover, 47% of intermediates demonstrated decreased bioaccumulation potential, 53% exhibited reduced mutagenicity, and 47% showed attenuated acute toxicity (Fig. S30b–d). The maximal reductions in these indices reached 82.22%, 100%, and 60.62%, respectively.

These comprehensive ecotoxicological evaluations unequivocally demonstrate that the Co_1_/C_3_N_5_/PMS system not only facilitates efficient OTC degradation but also yields transformation products with substantially lower environmental hazards and minimal potential for secondary pollution. The intrinsic selectivity and environmental compatibility of this catalytic system underscore its strong applicability for advanced wastewater treatment with low ecological risk.

## CONCLUSIONS

In this work, we developed a SAC, Co_1_/C_3_N_5_, via a coordination–recrystallization–argon pyrolysis method, featuring atomically dispersed Co centers initially coordinated in a Co–N_2_O_1_ configuration. This catalyst exhibited outstanding activity in PMS-based degradation of OTC, achieving a 5.3-fold enhancement over pristine C_3_N_5_ and outperforming over 30 previously reported catalysts. Beyond high activity, Co_1_/C_3_N_5_ demonstrated excellent durability under diverse water matrices, broad pH conditions (3.0–10.0), and repeated cycling, underscoring its robustness for practical water treatment.

Mechanistic investigations combining EPR spectroscopy, radical scavenging experiments, and DFT calculations revealed that ^1^O_2_, ·O_2_⁻, and ·SO_4_⁻ were the principal ROS. Notably, structural characterizations before and after reaction uncovered an *in-situ* coordination evolution from Co–N_2_O_1_ to Co–N_1_O_2_ during PMS activation. This transformation was found to govern the spin-state transition of Co, which in turn promoted a selective pathway for ^1^O_2_ generation. The dynamic restructuring of the Co coordination environment, together with its spin-sensitive activation behavior, underpins a previously unrecognized strategy for modulating the selectivity of PMS activation at single-atom sites.

This work not only highlights the catalytic potential of Co_1_/C_3_N_5_ for efficient and selective AOPs but also provides mechanistic insights into spin-regulated PMS activation driven by coordination environment dynamics, offering a paradigm for designing next-generation SACs for advanced water purification.

## MATERIALS AND METHODS

The catalyst synthesis, characterizations, performance evaluation, PMS concentration determination, and DFT calculations are discussed in *Supplementary Information*.

### Performance evaluation

In the OTC degradation experiments, an amount of catalyst was suspended in 20 mL of pollutant solution and stirred for 30 min to achieve adsorption–desorption equilibrium. Subsequently, a quantitative amount of PMS was added to the suspension to initiate the catalytic oxidation reaction. A volume of 1 mL of reaction solution was withdrawn at regular intervals and mixed with 1 mL of Na_2_S_2_O_3_ solution (1 mmol L^−1^) for quenching. The catalyst was separated by filtration using a hydrophilic PTFE membrane syringe filter (0.22 micron). Contaminant concentrations were monitored using a UV-visible spectrophotometer.

Recyclability studies were performed with the optimized catalyst (Co_1_/C_3_N_5_  *x*%). After each cycling experiment, the used catalyst was thoroughly washed with ethanol and deionised water and then used for the next degradation experiment. To obtain the regenerated catalyst, the cleaned catalyst was calcined at 300°C for 3 h. This regeneration process enables the catalyst to be reused for up to six consecutive cycles with good performance.

## Supplementary Material

nwag334_Supplemental_File
