# Supplementary material for: Spin-selection-favored peroxymonosulfate activation on reaction-induced Co–N1O2 sites enables 1O2-biased yet radical-parallel oxidation
Source: Natl Sci Rev. 2026 Jun 2;13(16):nwag334. doi: 10.1093/nsr/nwag334 (PMC13532579; doi:10.1093/nsr/nwag334)
Supplement: Supplementary file 1 [file nwag334_supplemental_file.pdf]

SUPPLEMENTARY FILE

Spin-selection-favored peroxymonosulfate activation on reaction-induced Co–N_1O_2 sites enables $^1O_2$-biased yet radical-parallel oxidation

Junlei Zhang[1], Guojia Yu[1], Qingqing Zhang[1], Haoran Wei[1], Wei Zhao[2], Shijie Li[3,*], Zhiyao Duan[1,*], and Sihui Zhan[4,*]

[1] School of Materials Science and Engineering, Northwestern Polytechnical University, Xi'an 710072, P.R. China

[2] School of Ecology and Environmental Science, Institute for Ecological Research and Pollution Control of Plateau Lakes, Yunnan University, Kunming 650500, P.R. China

[3] National Engineering Research Center for Marine Aquaculture, Zhejiang Key Laboratory of Petrochemical Environmental Pollution Control, Zhejiang Ocean University, Zhoushan 316000, P.R. China

[4] School of Environmental Science & Engineering, Tianjin University, Tianjin 300350, P.R. China

[*]Corresponding authors. E-mails: zhiyao.duan@nwpu.edu.cn; lishijie@zjou.edu.cn; sihuizhan@gmail.com

SUPPORTING INFORMATION TEXT

MATERIALS AND METHODS

1. Catalyst synthesis

Co_1/C_3N_5 was synthesised by a simple one-step pyrolysis process following a coordination-recrystallisation strategy in a tube furnace. 1.6 g of 3-amino-1,2,4-triazole was dissolved in 10 mL of deionised water. Then, different volumes (X = 0.25, 2 or 3 mL) of Co^{2+} solution (1 mmol/mL) were added dropwise to the above solution with constant stirring for 4 hours to ensure complete coordination between Co^{2+} and 3-amino-1,2,4-triazole. The final mixture was subjected to a recrystallisation step at 99 °C for 48 hours. The Co_1/C_3N_5 samples were then pyrolysed in a tube furnace at 550 °C for 3 hours in Ar. The solid obtained was ground into a fine powder for subsequent applications. According to our previous study, a pure C_3N_5 sample was synthesised by calcining 1.6 g of 3-amino-1,2,4-triazole in a tube furnace at 550 °C for 3 h in Ar.

2. PMS concentration determination

The concentration of PMS was measured by spectrophotometry. The mixture (0.01 g of $NaHCO_3$ + 0.2 g of KI+ 0.3 mL of Sample + 4.0 mL of DIW) was reacted for 10 min and the absorbance was measured at 352 nm.

3. Density functional theory (DFT) calculations

In this work, the Vienna Ab initio Simulation Package (VASP) was utilized to perform density functional theory (DFT) calculations based on a plane wave basis [1, 2]. The Perdew-Burke-Ernzerhof (PBE) functional was chosen to describe the electronic exchange and correlation effects [3]. Core electrons were represented within the projector augmented wave (PAW) method [4]. The plane wave basis was set with a cut-off energy of 400 eV. For the electronic relaxation and self-consistent field calculations, the convergence criteria were both set to 10^{-5} eV. The atomic structures were considered fully optimized when the residual forces each on atom were reduced below 0.05 eV/Å.

A C_3N_5 surface model based on the xy plane was employed to simulate reaction processes, where all reactions were carried out on the C_3N_5 surface. To avoid interactions between periodic images, a vacuum layer of at least 25 Å was applied along the z-axis. The Brillouin zone integration in k-space was performed using a 2 × 2 × 1 gamma-centered k-point mesh.

The adsorption energy (E) has been calculated using **Eqs. 1**. The Gibbs free energy change (AG) of each chemical reaction was calculated by **Eqs. 2**.

$$E_{ads}=E_{total}-E_{substrate}-E_{adsorbate} \quad (1)$$

The E_{total}, $E_{substrate}$ and $E_{adsorbate}$ represent the energy of adsorption structure, slab ($Co_3B(103)/Co_3O_4(110)$) and adsorbate (PMS molecule), respectively.

The Gibbs free energy change (ΔG) of each chemical reaction was calculated by Eq. 2,

$$\Delta G=\Delta E+\Delta ZPE-T\Delta S \quad (2)$$

where E is the calculated total energy, ZPE is the zero-point energy, T is the temperature, and S is the entropy.
In VASP calculations, magnetic moments of different atoms were evaluated using the LORBIT = 10 tag. And the nudged elastic band (NEB) methodology is recognized as an efficient algorithm for calculating reaction activation energies.

4. Characterizations measurements

X-ray diffraction (XRD) tests were performed on a X'Pert PRO MPD X-ray diffractometer with Cu Kα radiation at 30 kV and 30 mA at a scanning speed of 8°/min. X-ray photoelectron spectroscopy (XPS) data were collected by a multifunctional photoelectron spectroscopy instrument (K-Alpha+, Thermo). High-angle annular dark field-scanning TEM images (HAADF-STEM) were collected on JEOL ARM-200F transmission electron microscope with an accelerating voltage of 200 kV. X-ray absorption fine structure (XAFS) spectroscopy was carried out using the RapidXAFS 2M (Anhui Absorption Spectroscopy Analysis Instrument Co., Ltd.) by transmission（or fluorescence） mode at 20 kV and 20 mA, and the Si (551) spherically bent crystal analyzer with a radius of curvature of 500 mm was used for Co.

SUPPORTING INFORMATION FIGURES

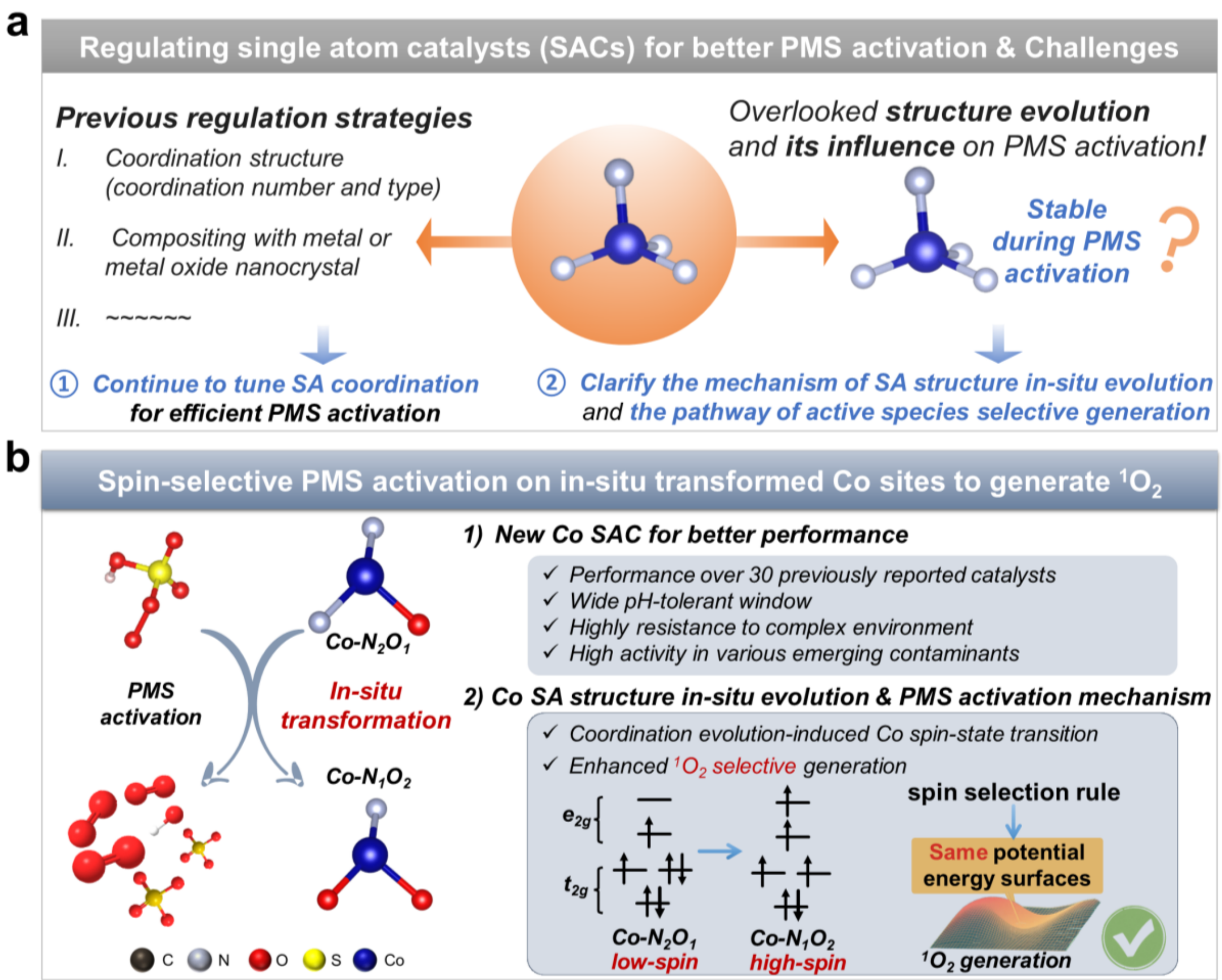

Figure S1. (a) Progress and challenges in regulating single atom catalysts (SACs) for peroxymonosulfate (PMS) activation; (b) This work: spin-selective PMS activation via in situ-generated Co–N_1O_2 atomic sites for efficient degradation of emerging contaminants (ECs).

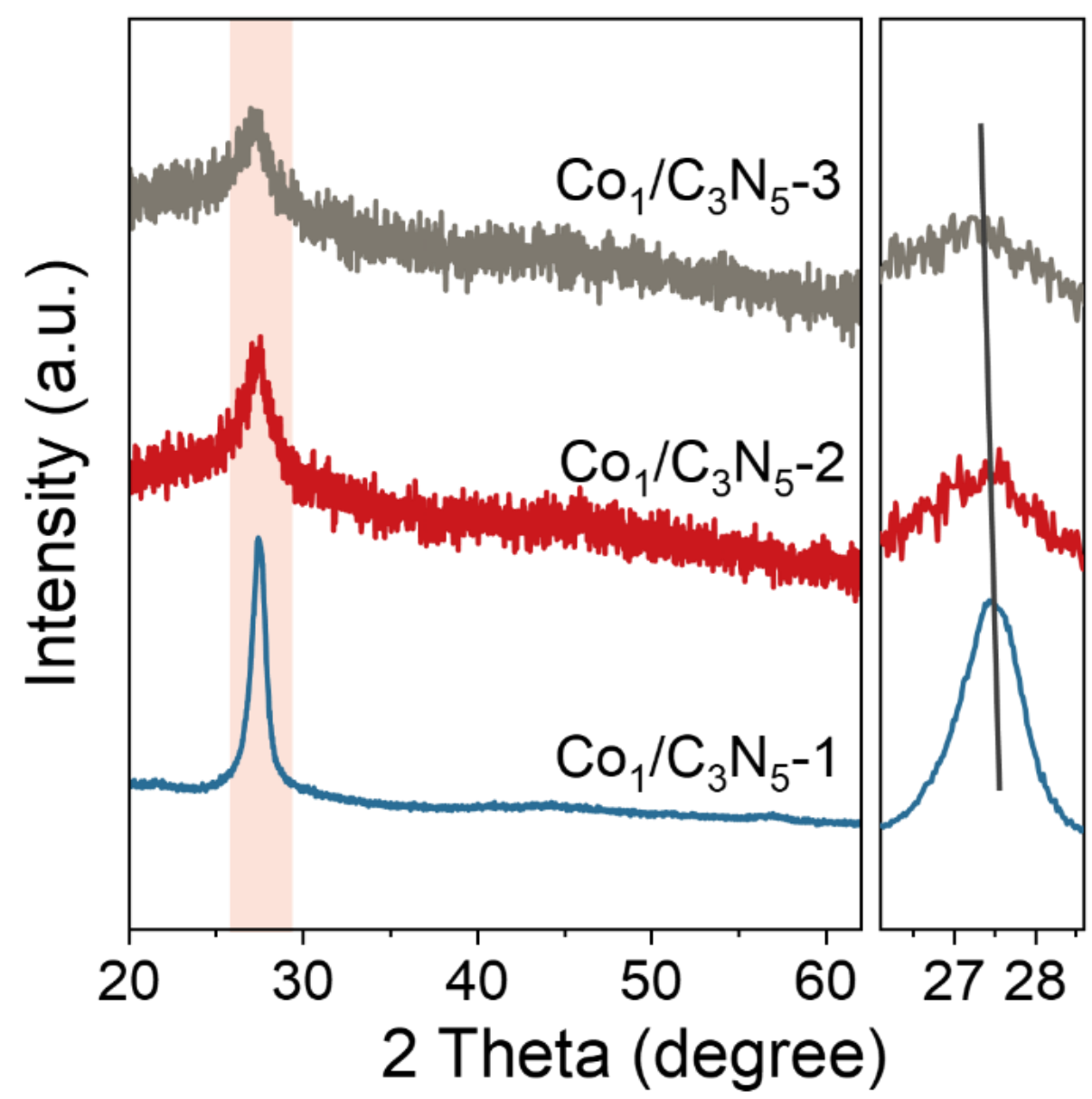

Figure S2. X-ray diffraction (XRD) patterns of Co_1/C_3N_5-1, Co_1/C_3N_5-2 and Co_1/C_3N_5-3.

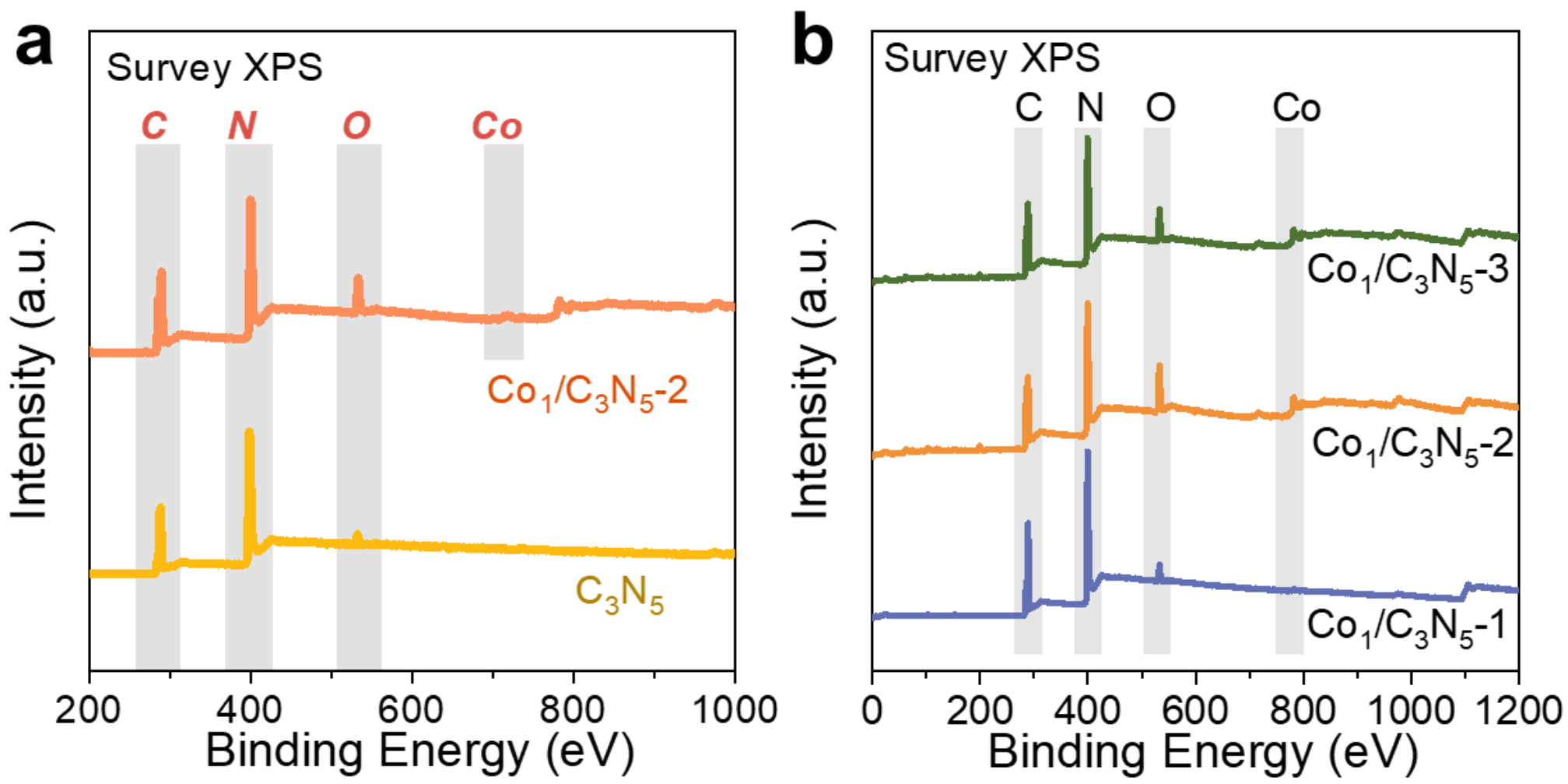

Figure S3. (a-b) Survey high-resolution X-ray photoelectron spectroscopy (XPS) of C_3N_5, Co_1/C_3N_5-1, Co_1/C_3N_5-2 and Co_1/C_3N_5-3.

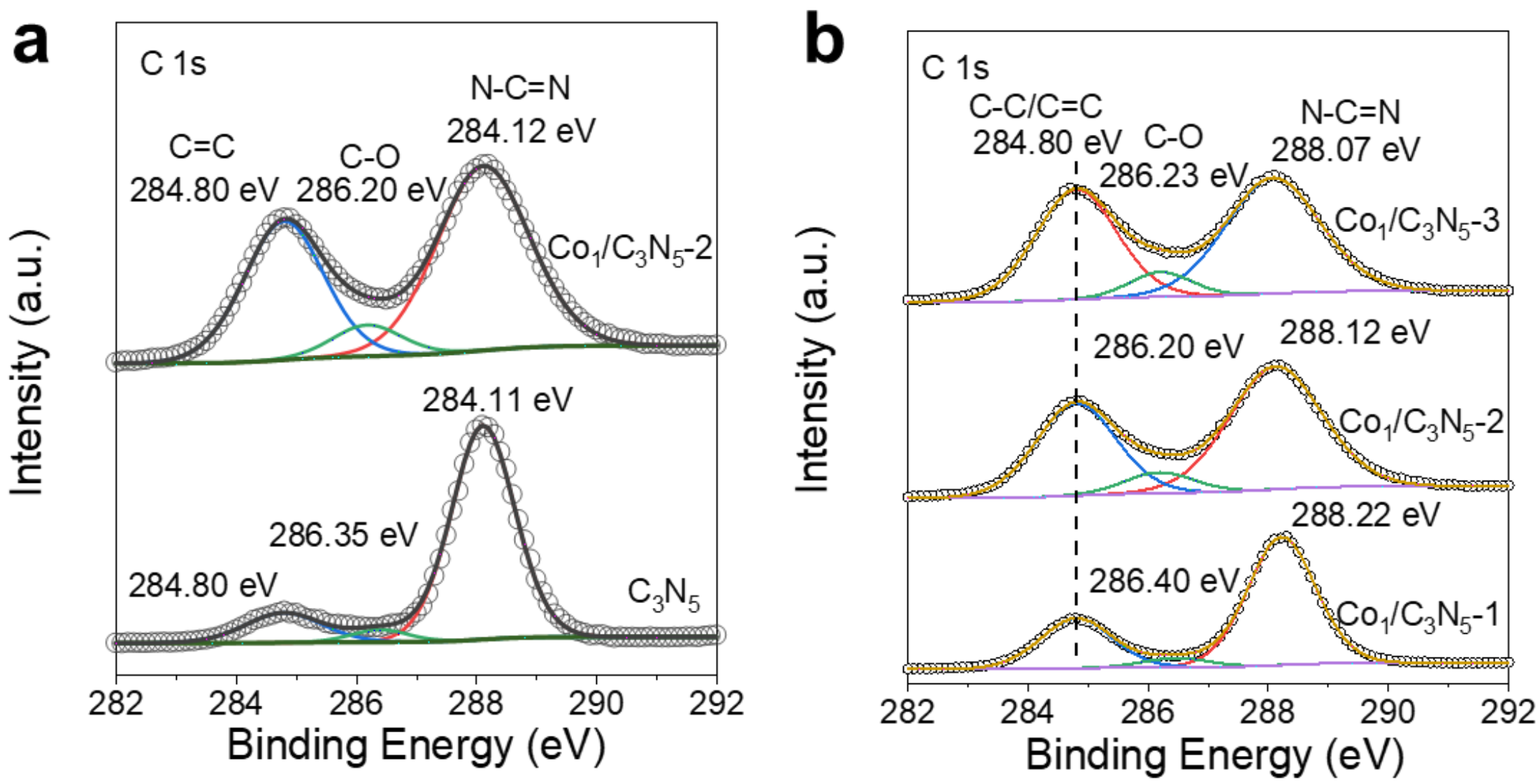

Figure S4. (a-b) C 1s XPS spectra of C_3N_5, Co_1/C_3N_5-1, Co_1/C_3N_5-2 and Co_1/C_3N_5-3.

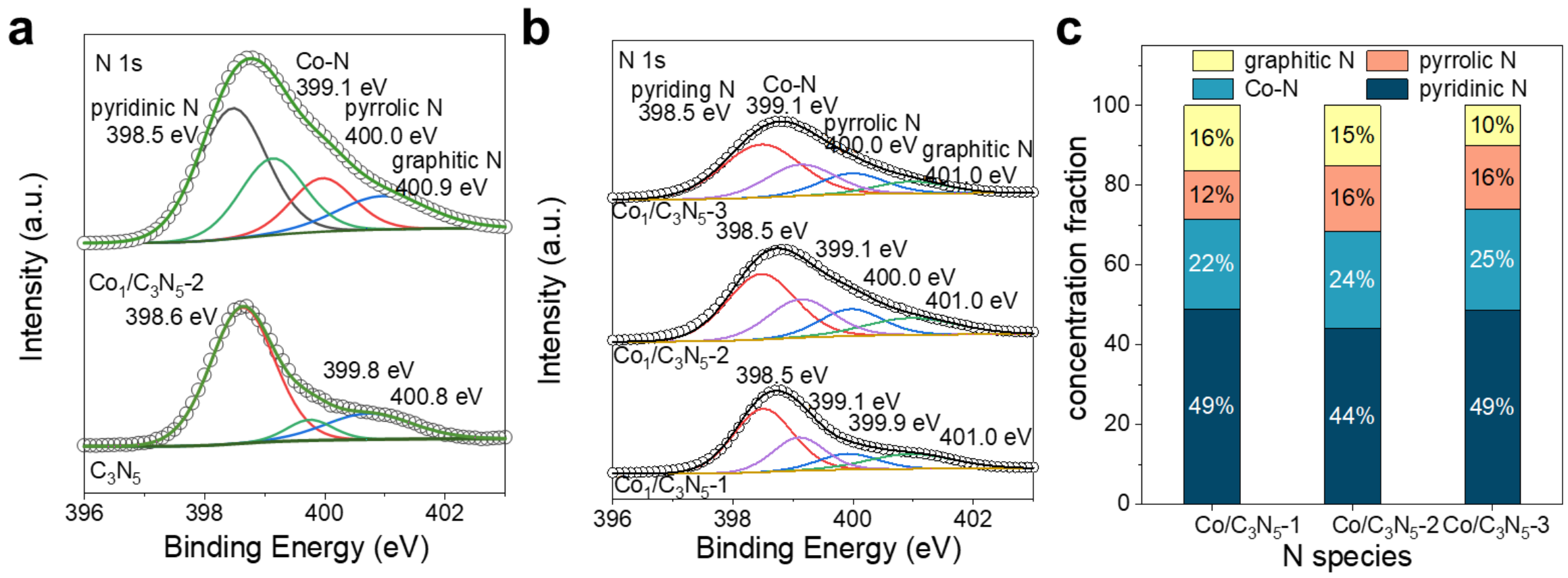

Figure S5. (a-b) N 1s XPS spectra of C_3N_5, Co_1/C_3N_5-1, Co_1/C_3N_5-2 and Co_1/C_3N_5-3. (c) The relative abundance (%) of various N functional groups on C_3N_5, Co_1/C_3N_5-1, Co_1/C_3N_5-2 and Co_1/C_3N_5-3.

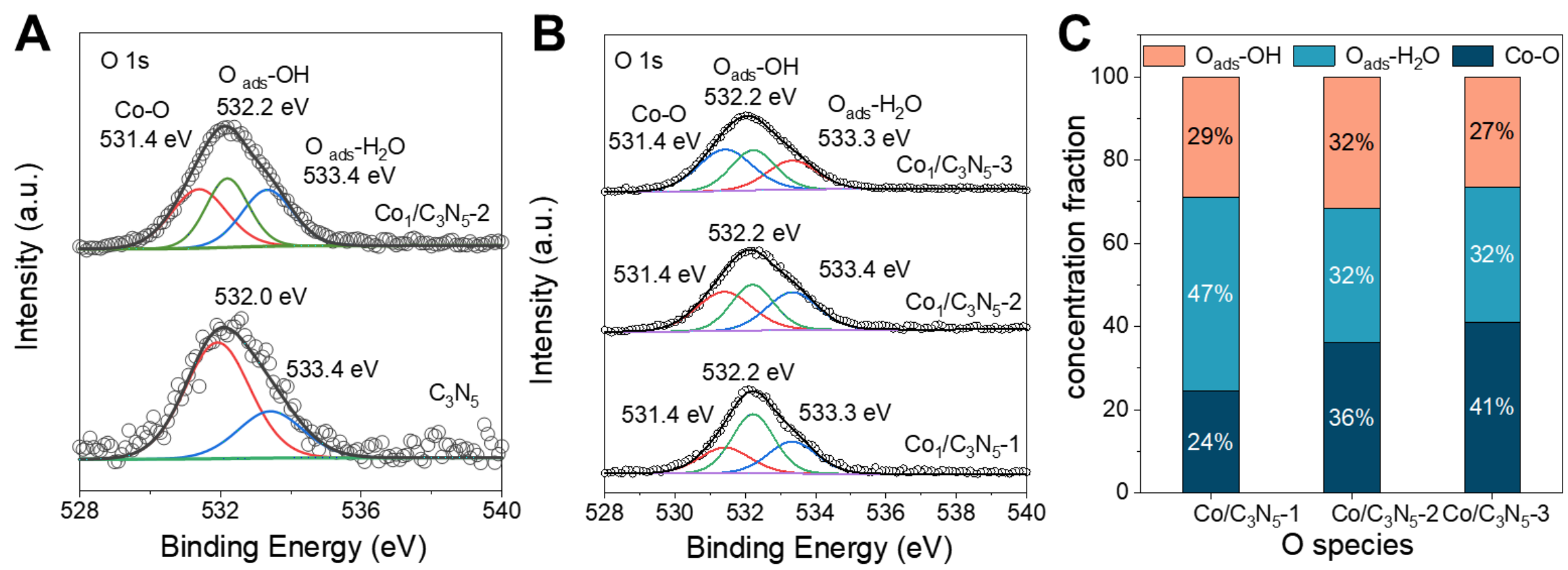

Figure S6. (a-b) O 1s XPS spectra of C_3N_5, Co_1/C_3N_5-1, Co_1/C_3N_5-2 and Co_1/C_3N_5-3. (c) The relative abundance (%) of various O functional groups on C_3N_5, Co_1/C_3N_5-1, Co_1/C_3N_5-2 and Co_1/C_3N_5-3.

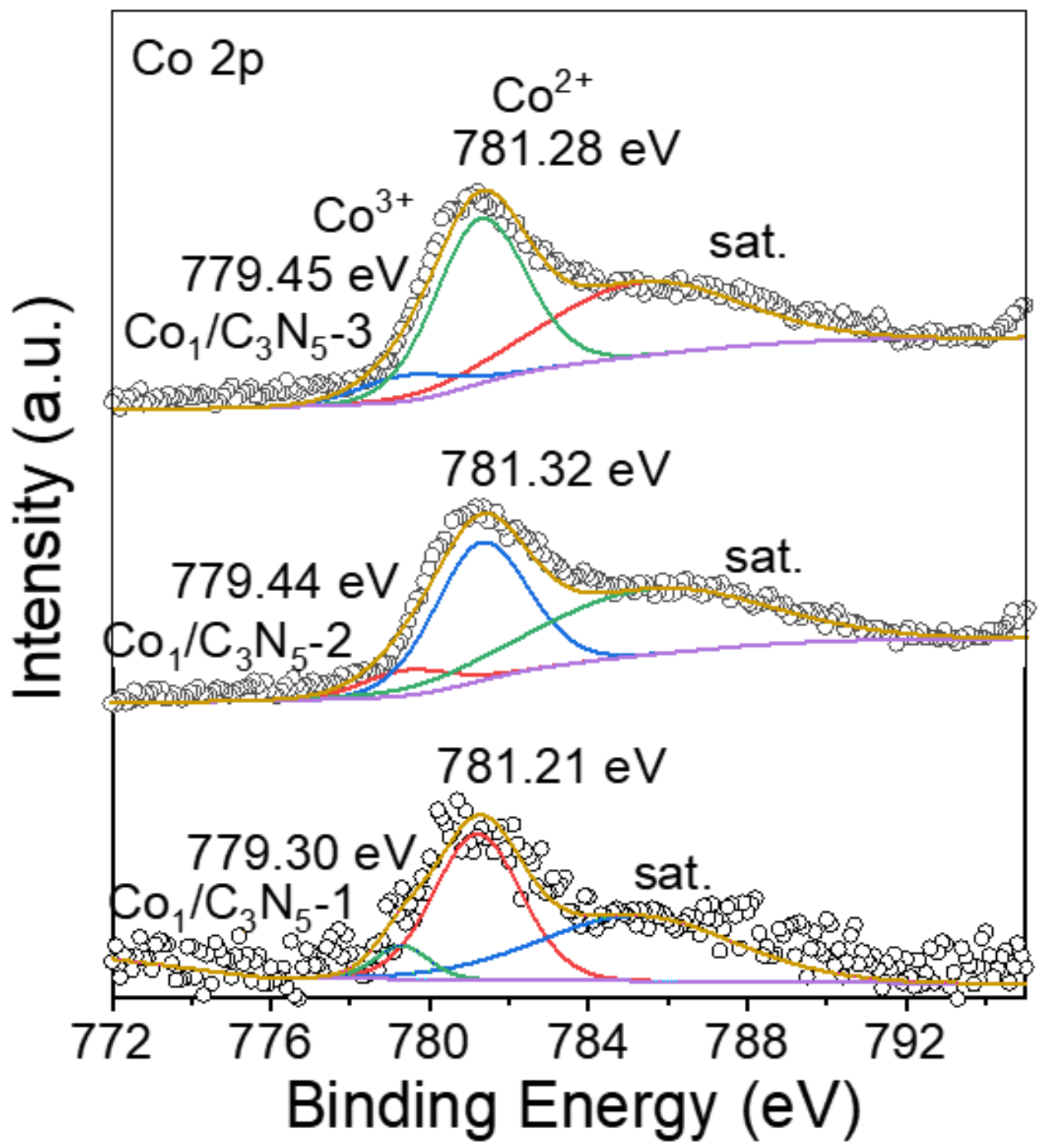

Figure S7. Co 2p XPS spectra of Co_1/C_3N_5-1, Co_1/C_3N_5-2 and Co_1/C_3N_5-3.

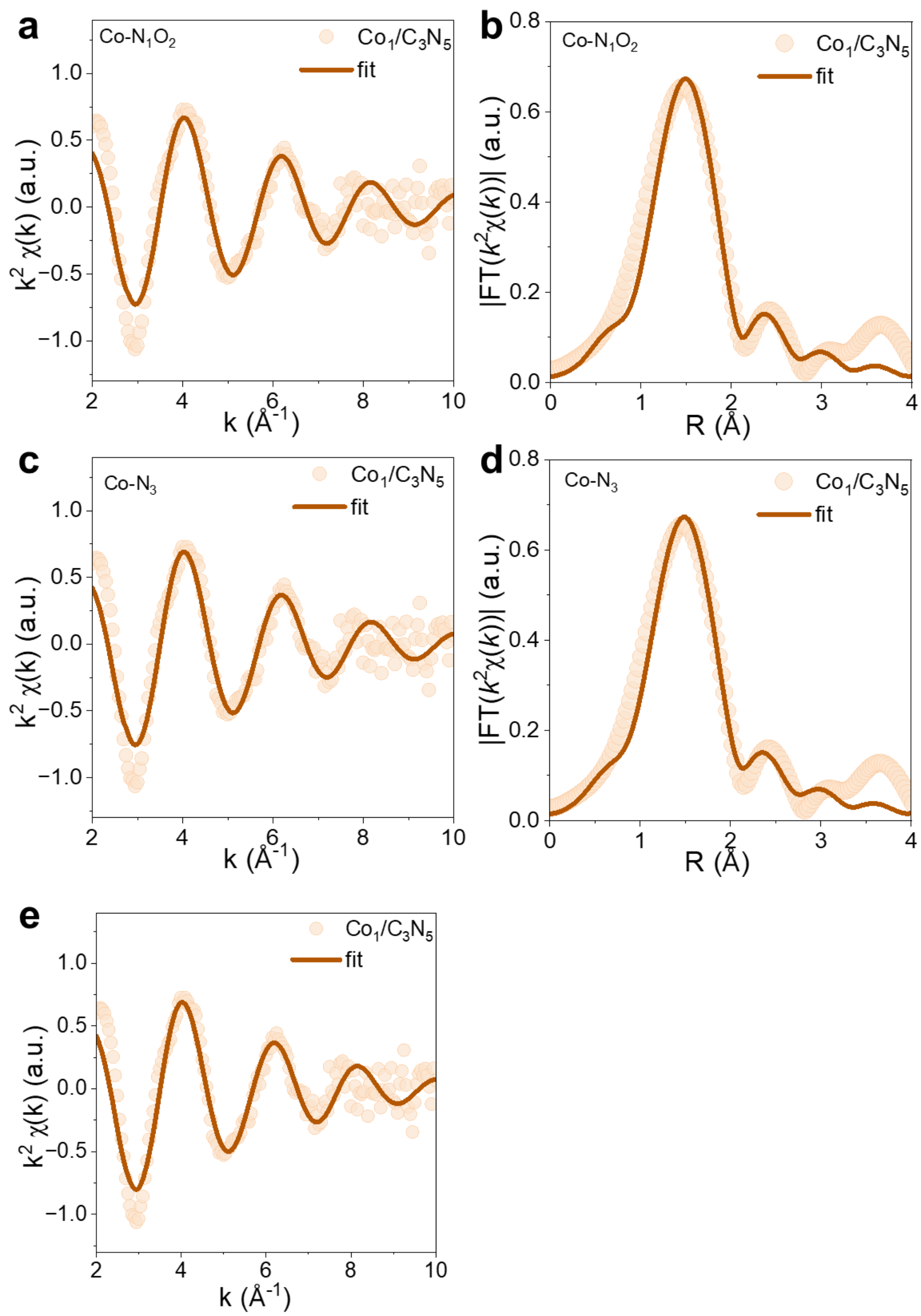

Figure S8. (a, b) The prediction of Fourier-transformed k^2-weighted EXAFS curve (k and R space) for Co-N_1O_2, (c, d) The prediction of Fourier-transformed k^2-weighted EXAFS curve (k and R space) for Co-N_3.

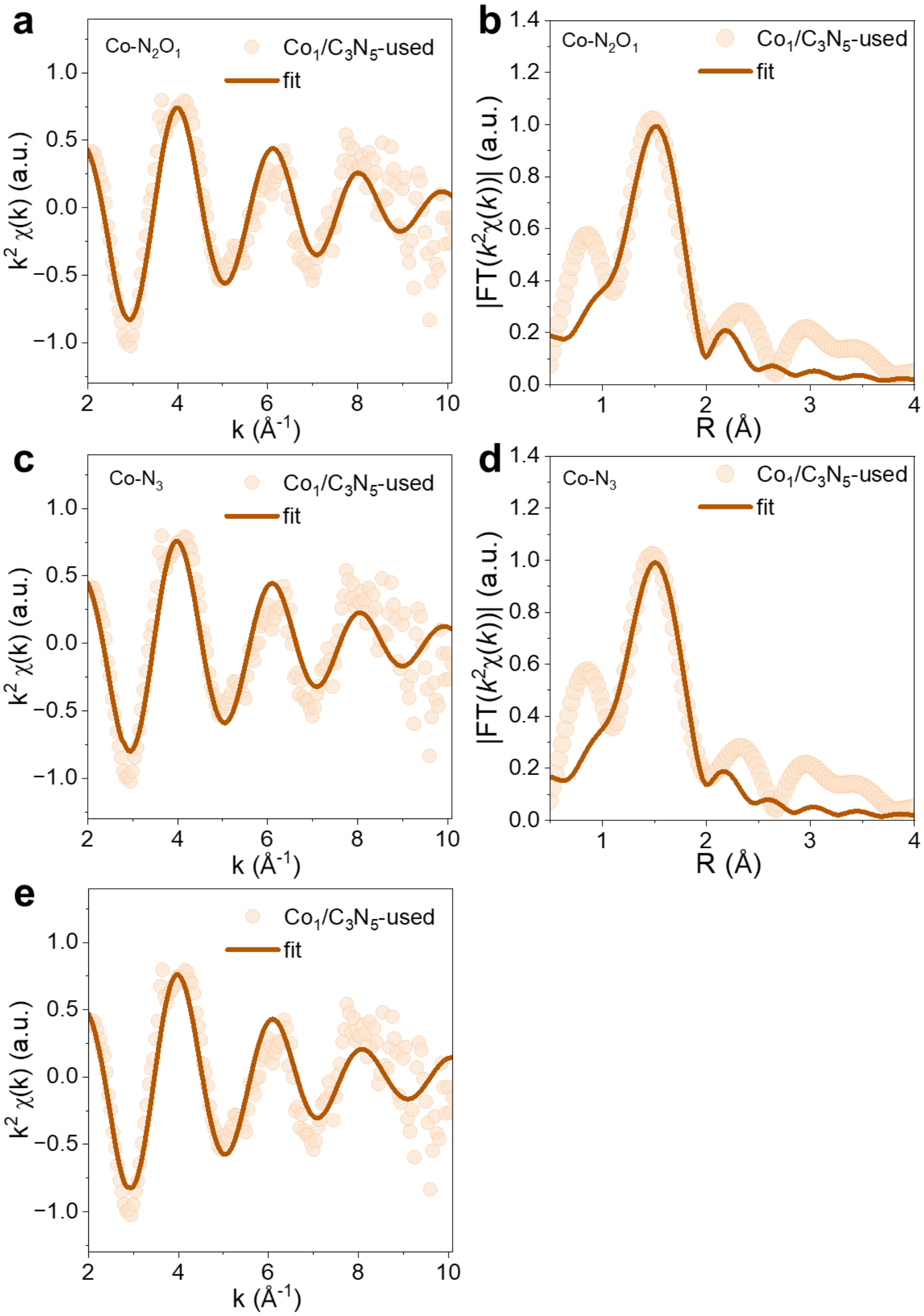

Figure S9. (a, b) The prediction of Fourier-transformed k^2-weighted EXAFS curve (k and R space) for $Co\text{-}N_2O_1$, (c, d) The prediction of Fourier-transformed k^2-weighted EXAFS curve (k and R space) for $Co\text{-}N_3$. (e) EXAFS fitting curves in K space of Co_1/C_3N_5-used for $Co\text{-}N_1O_2$.

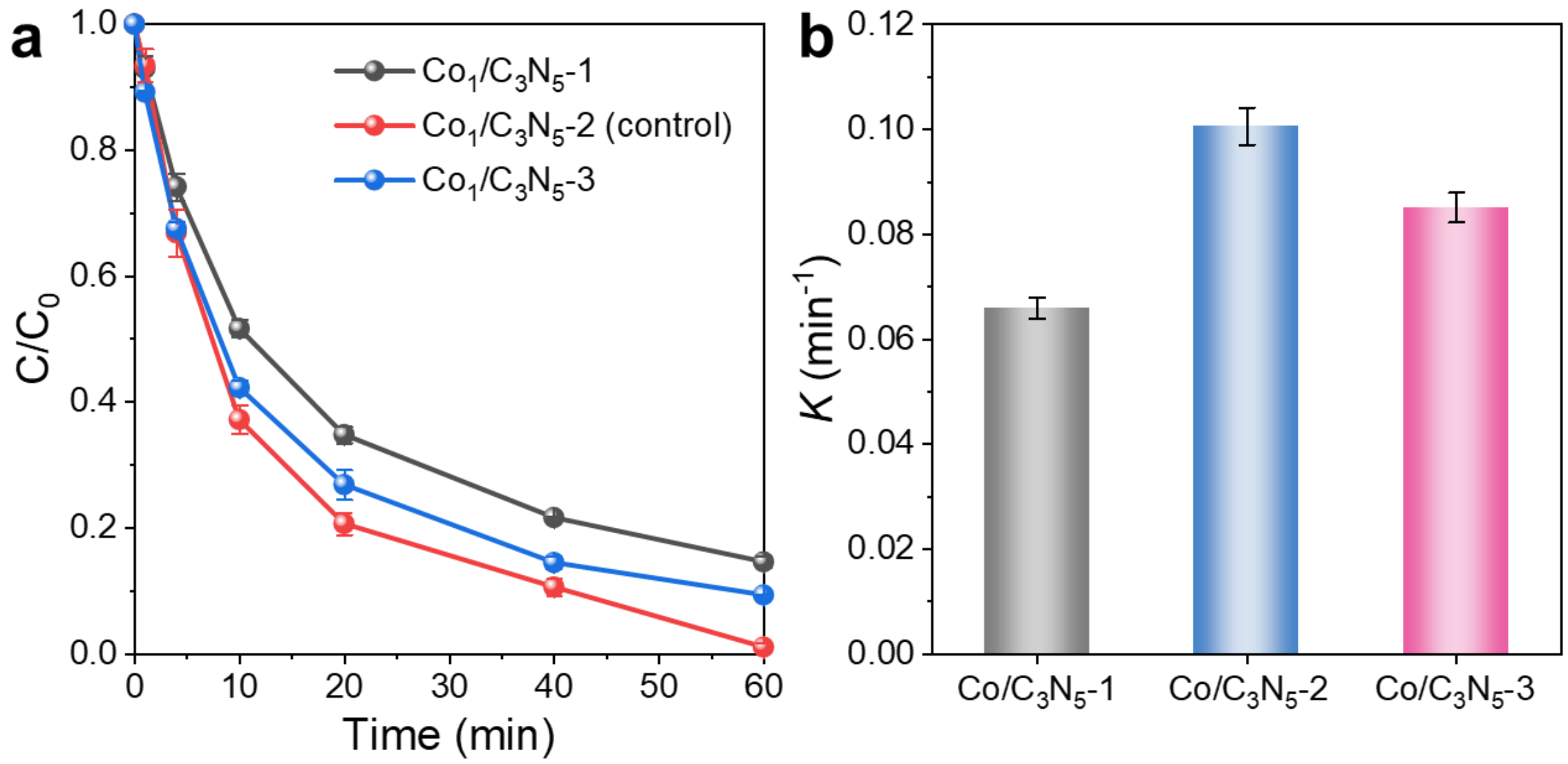

Figure S10. (a) Degradation curves of OTC. (b) Degradation rates of OTC.

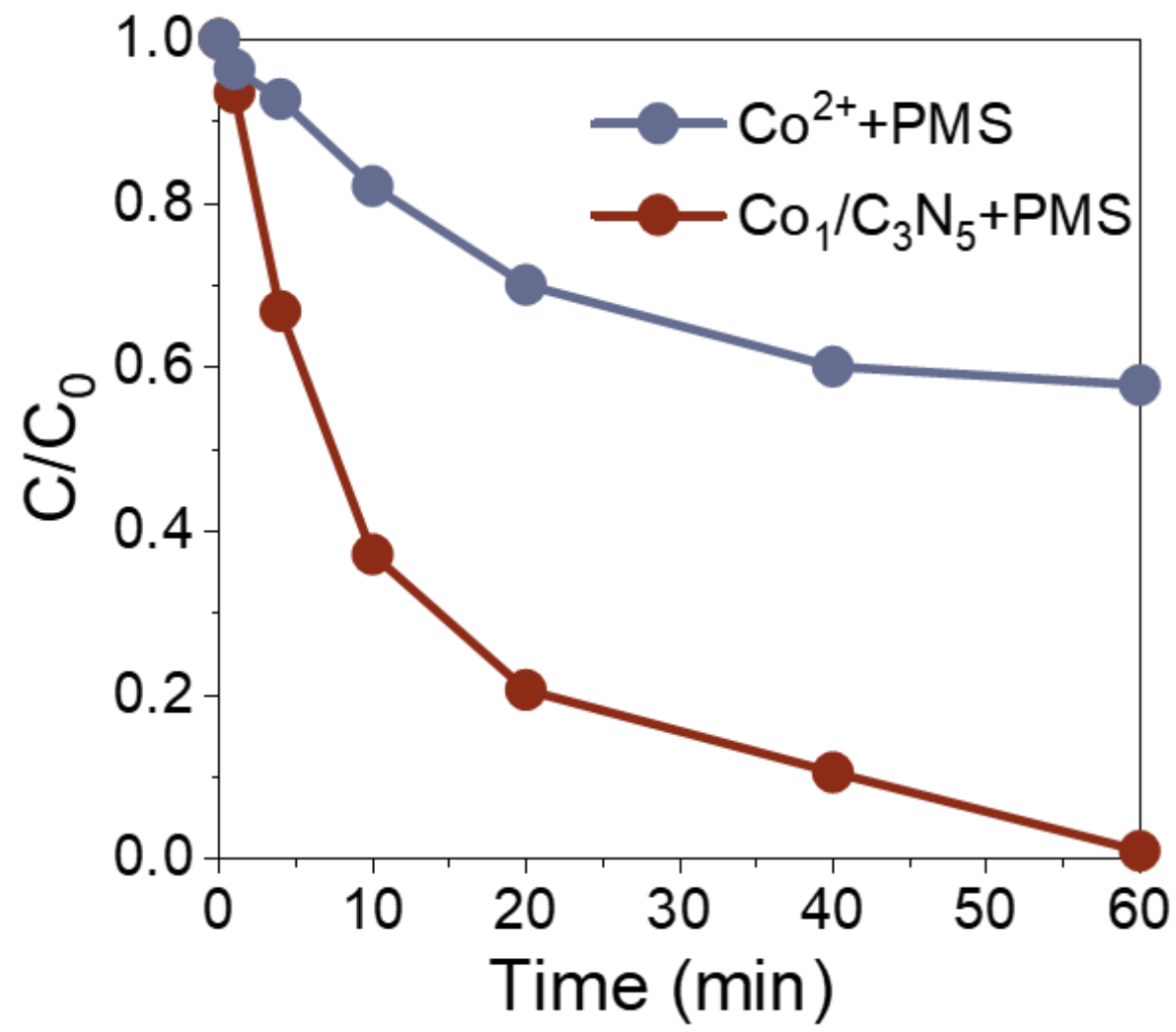

Figure S11. Degradation curves of OTC in the Co^{2+} coupled PMS system.

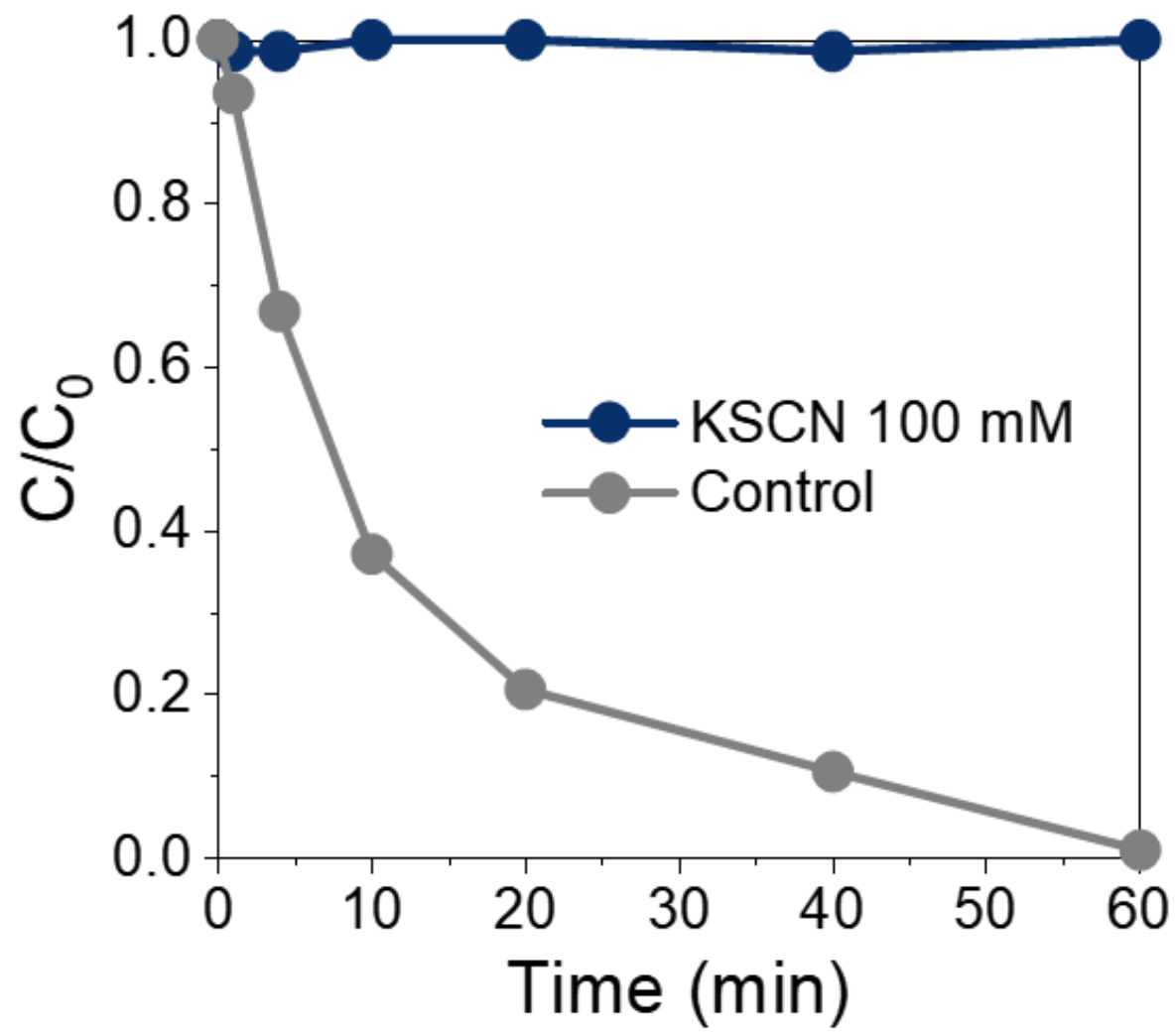

Figure S12. Comparison of the efficiency of OTC degradation in the Co_1/C_3N_5 coupled PMS system with and without potassium thiocyanate (KSCN).

According to previous report**[5]**, KSCN can form highly stable metal-ligand complexes with cobalt, thereby effectively inhibiting cobalt's participation in heterogeneous reactions. The experimental results demonstrated that the addition of KSCN nearly completely suppressed OTC degradation, which strongly suggests that Co sites serve as the dominant active centers for PMS activation in the Co_1/C_3N_5/PMS system.

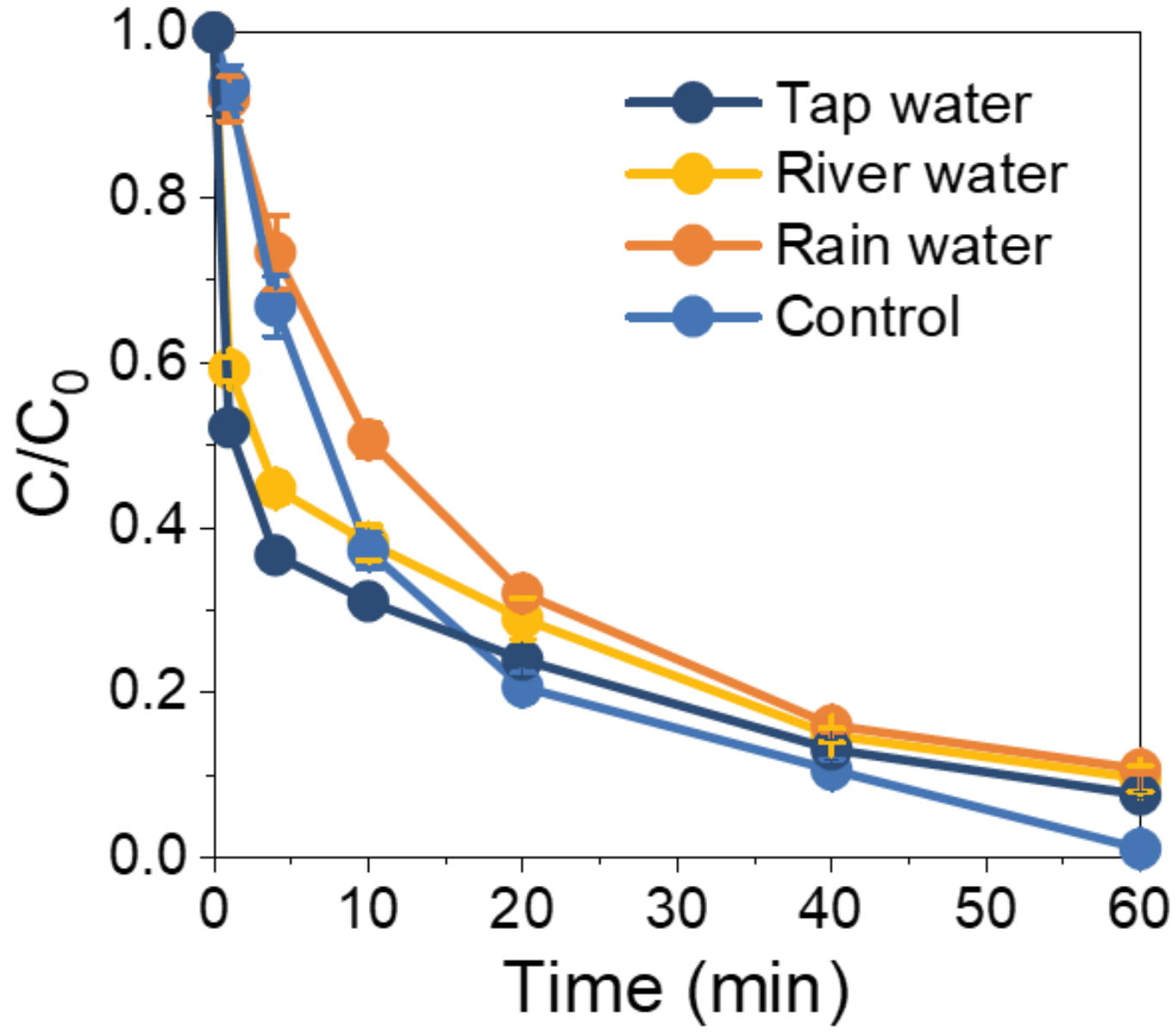

Figure S13. Influence of different water bodies on OTC degradation in the system using Co_1/C_3N_5 to activate PMS.

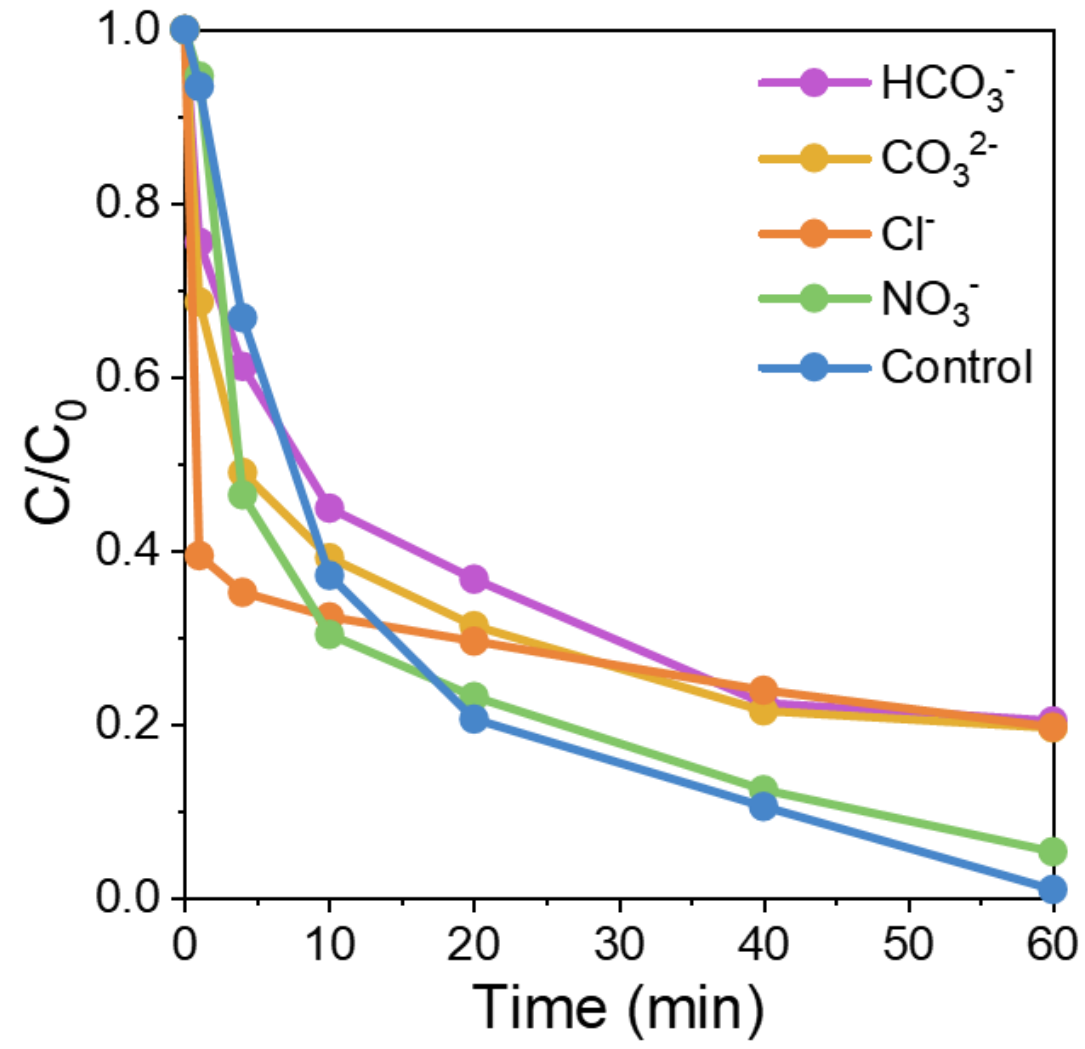

Figure S14. Influence of various anions on OTC degradation in the system using Co_1/C_3N_5 to activate PMS. (HCO_3^- 10 mM, CO_3^{2-} 10 mM, Cl^- 7 mM, and NO_3^- 10 mM).

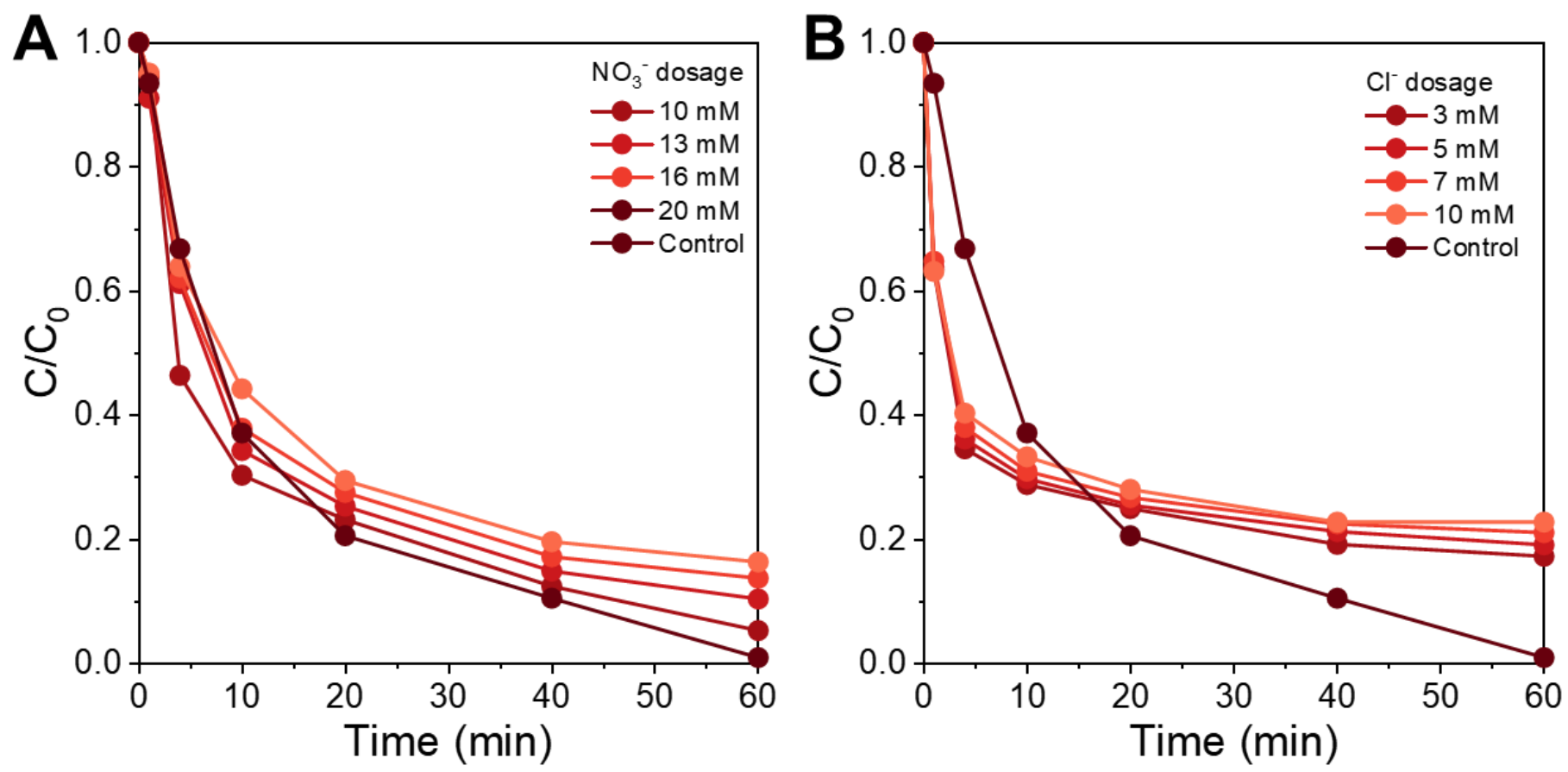

Figure S15. Influence of (a) NO_3^- dosage and (b) Cl^- dosage on OTC degradation in the system using Co_1/C_3N_5 to activate PMS.

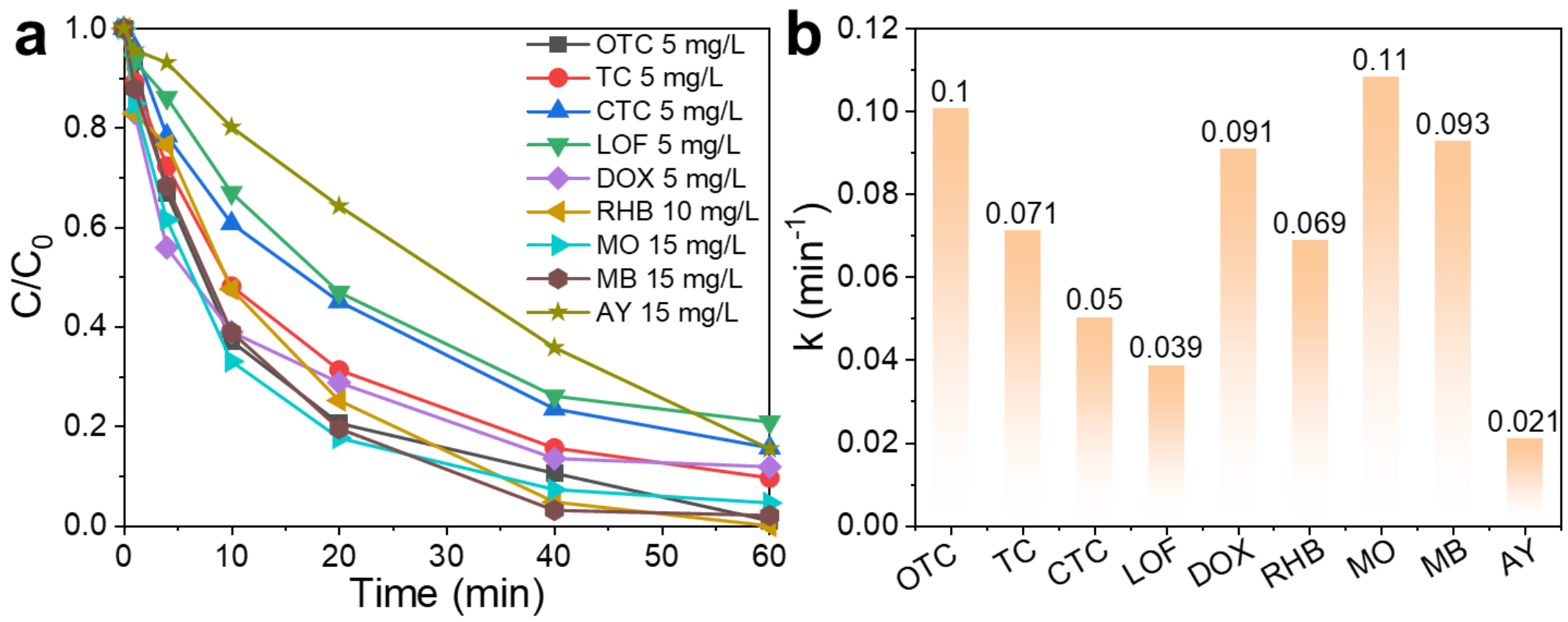

Figure S16. (a) Removal efficiency and (b) rate of various organic pollutants using Co_1/C_3N_5 to activate PMS.

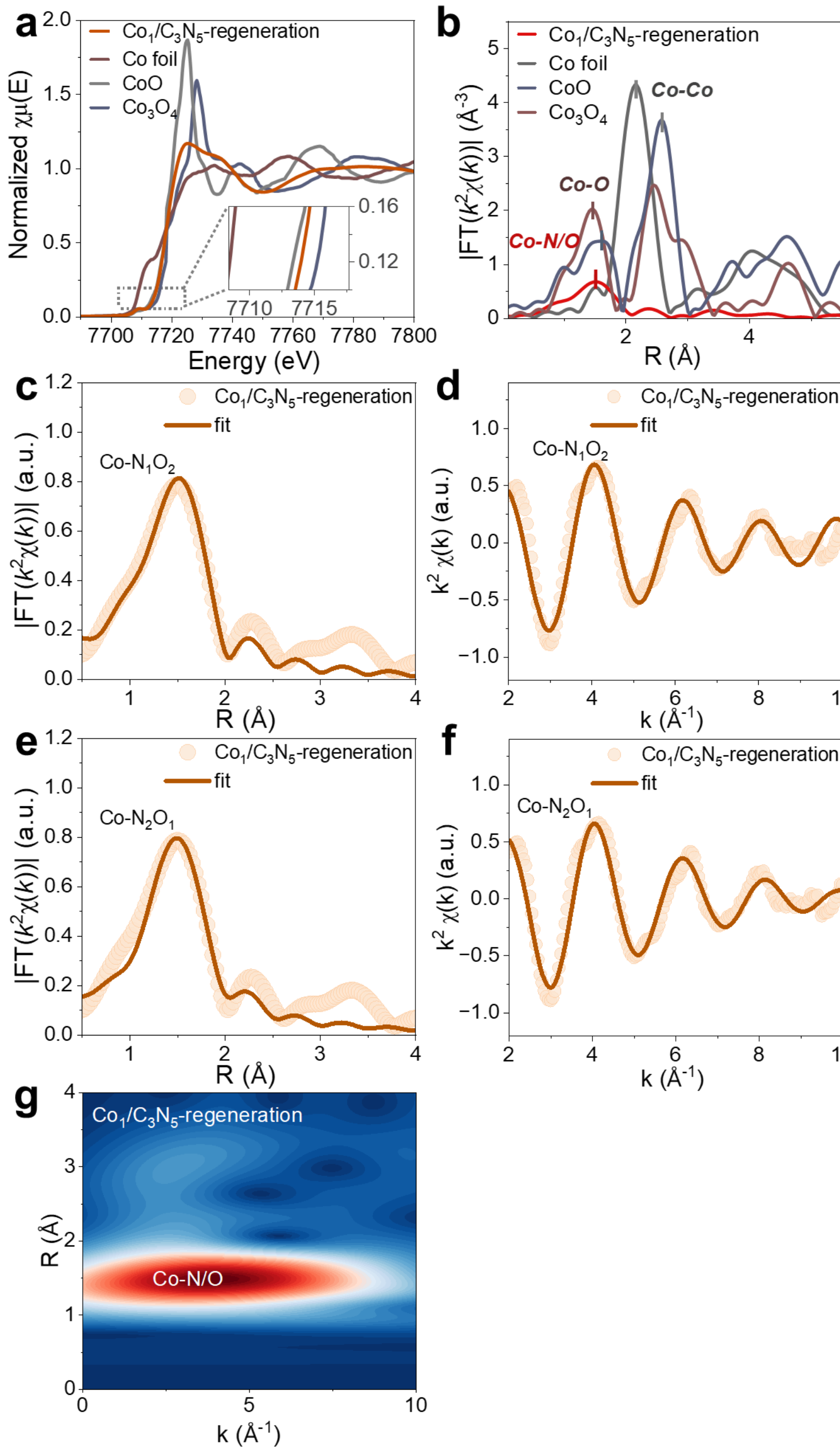

Figure S17. (a) Co K-edge X-ray absorption near-edge structure (XANES). (b) Fourier-transformed extended X-ray absorption fine structure (FT-EXAFS) without Co-Co signature. (c, d) The prediction of Fourier-transformed k^2-weighted EXAFS curve (k and R space) for Co-N_1O_2, (e, f) The prediction of Fourier-transformed k^2-weighted EXAFS curve (k and R space) for Co-N_2O_1. (g) Wavelet transform EXAFS.

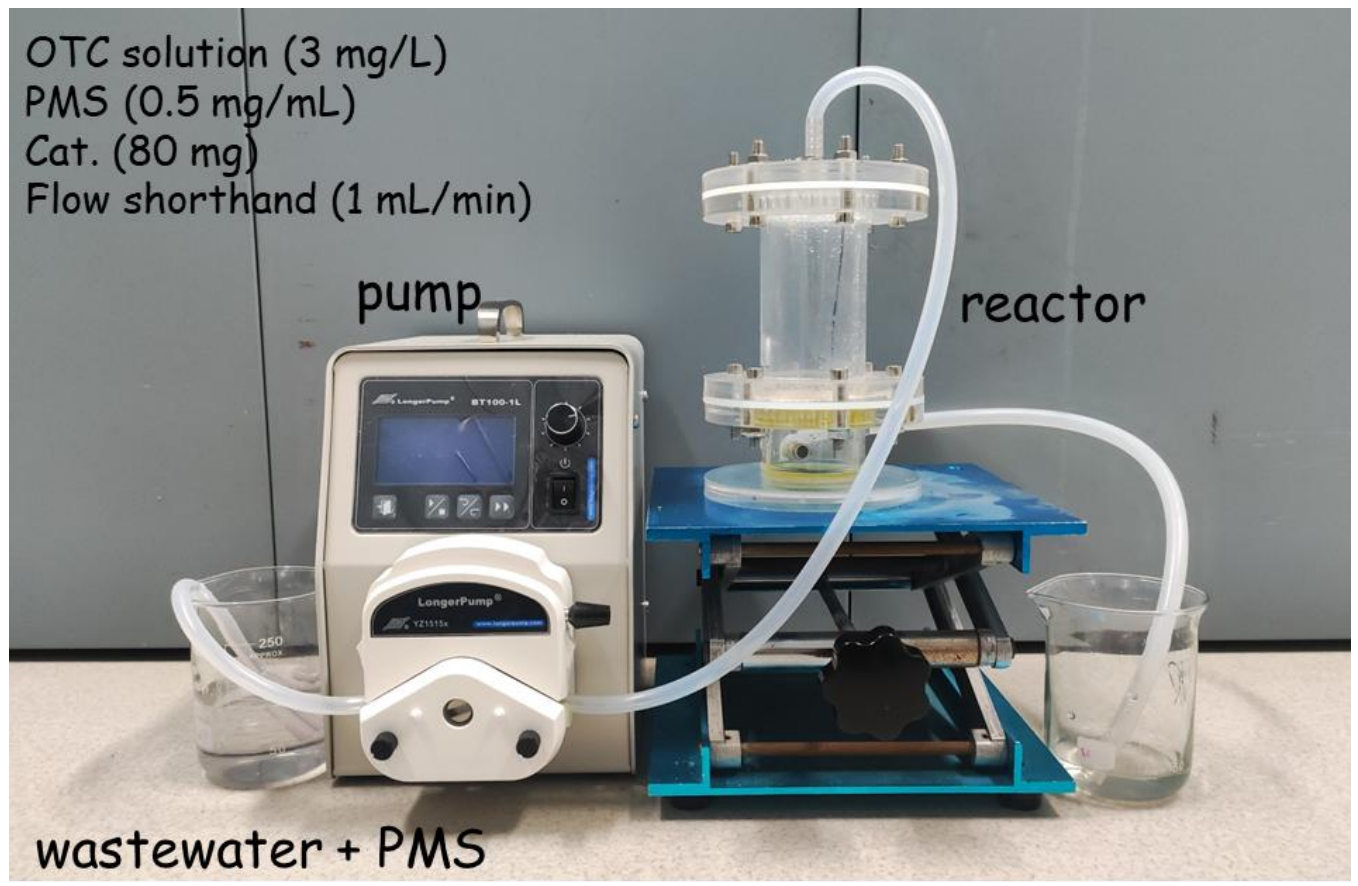

Figure S18. The wastewater treatment experiment equipment of simulated emergency wastewater by Co_1/C_3N_5.

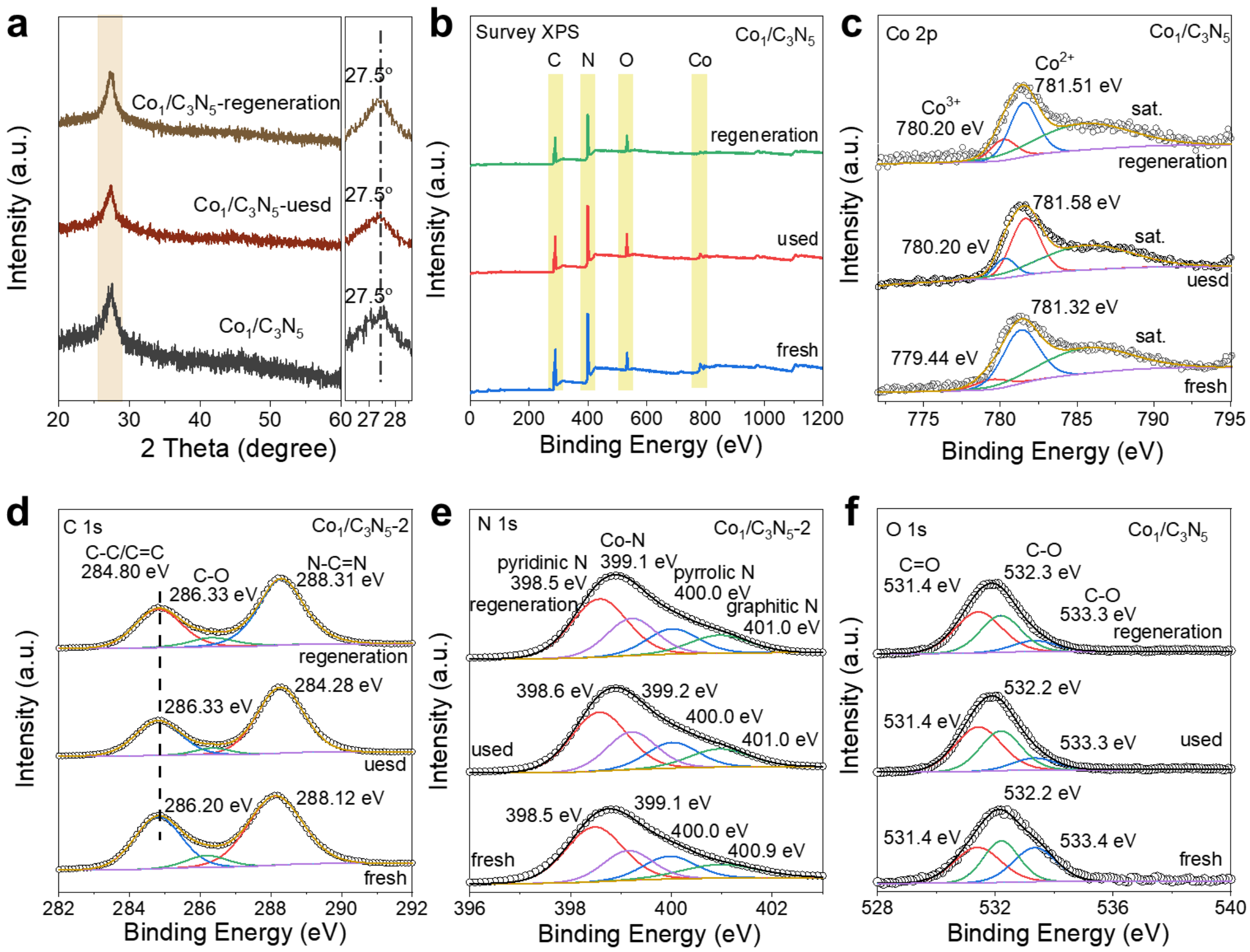

Figure S19. (a) XRD patterns of fresh Co_1/C_3N_5, spent Co_1/C_3N_5, and regenerated Co_1/C_3N_5. (b) Survey high-resolution X-ray photoelectron spectroscopy (XPS) of Co_1/C_3N_5, Co_1/C_3N_5-used and Co_1/C_3N_5-regeneration samples. (c-f) XPS spectra of Co $2p_{3/2}$, C 1 s, O 1 s, and N 1 s on Co_1/C_3N_5, Co_1/C_3N_5-used and Co_1/C_3N_5-regeneration samples.

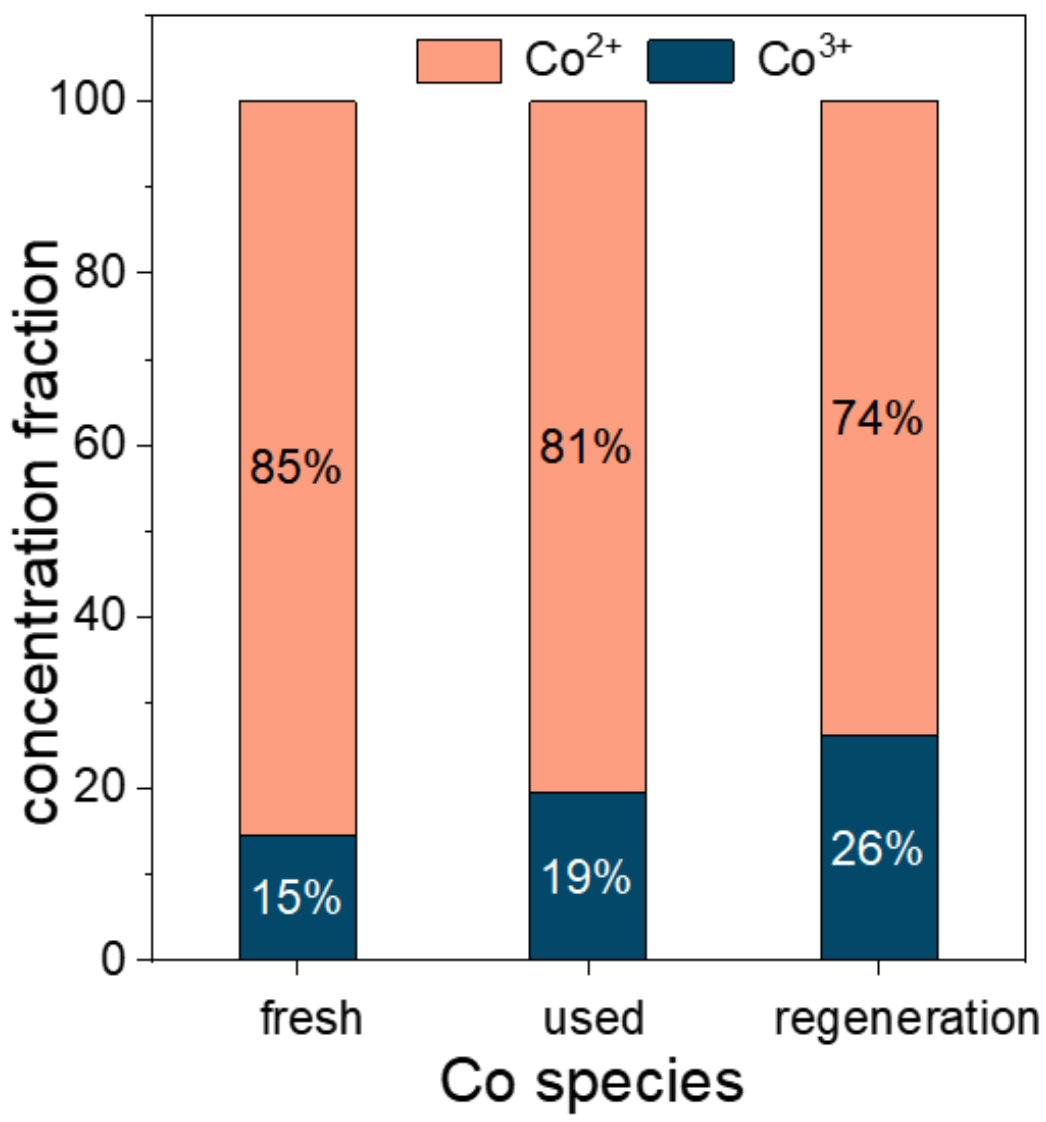

Figure S20. The relative abundance (%) of various Co^{2+}/Co^{3+} on Co_1/C_3N_5.

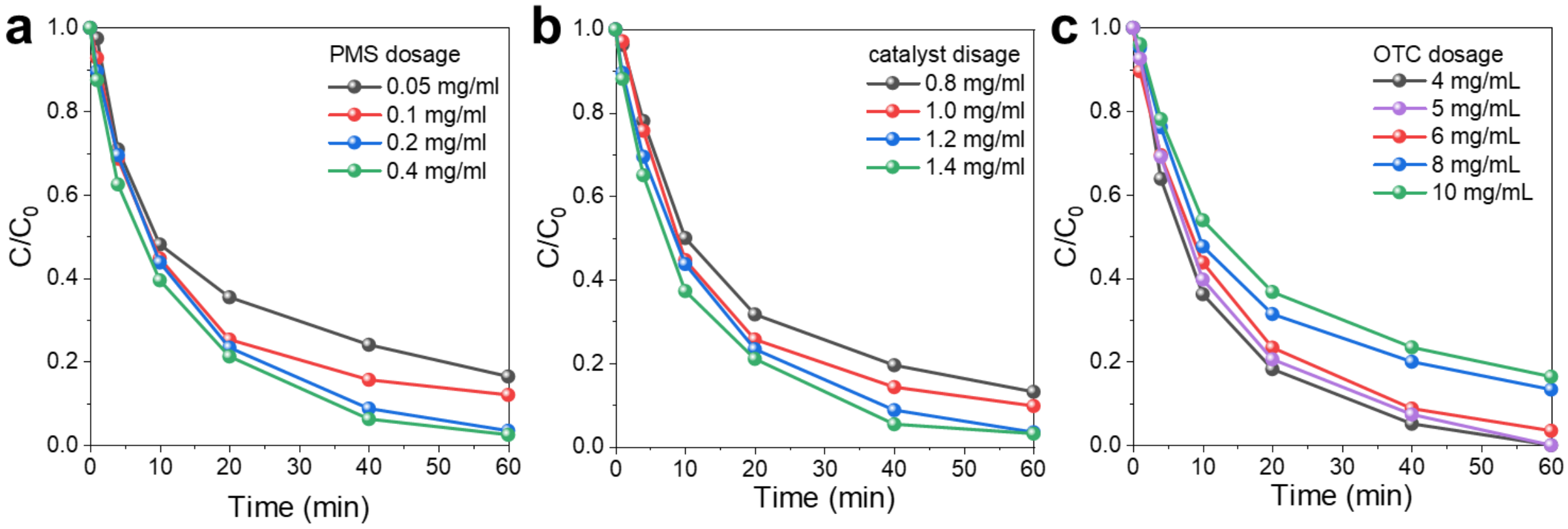

Figure S21. Influence of (a) PMS dosage (OTC 6 mg/L, catalyst 1.2 mg/ml), (b) catalyst dosage (OTC 6 mg/L, PMS 0.2 mg/ml) and (c) OTC dosage (catalyst 1.2 mg/ml, PMS 0.2 mg/ml) on OTC degradation in the system using Co_1/C_3N_5 to activate PMS.

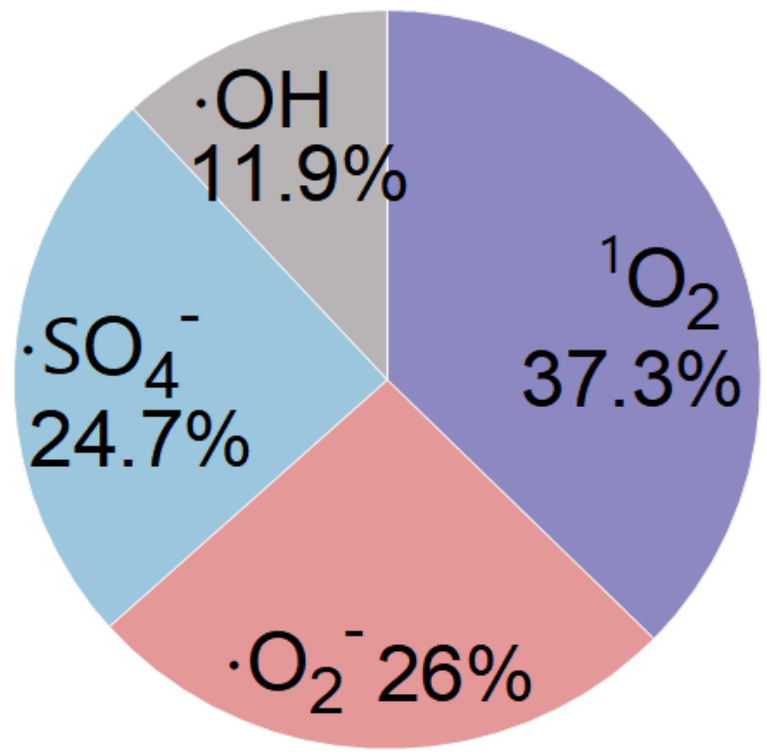

Figure S22. The relative contributions of radical and non-radical in Co_1/C_3N_5/PMS system.

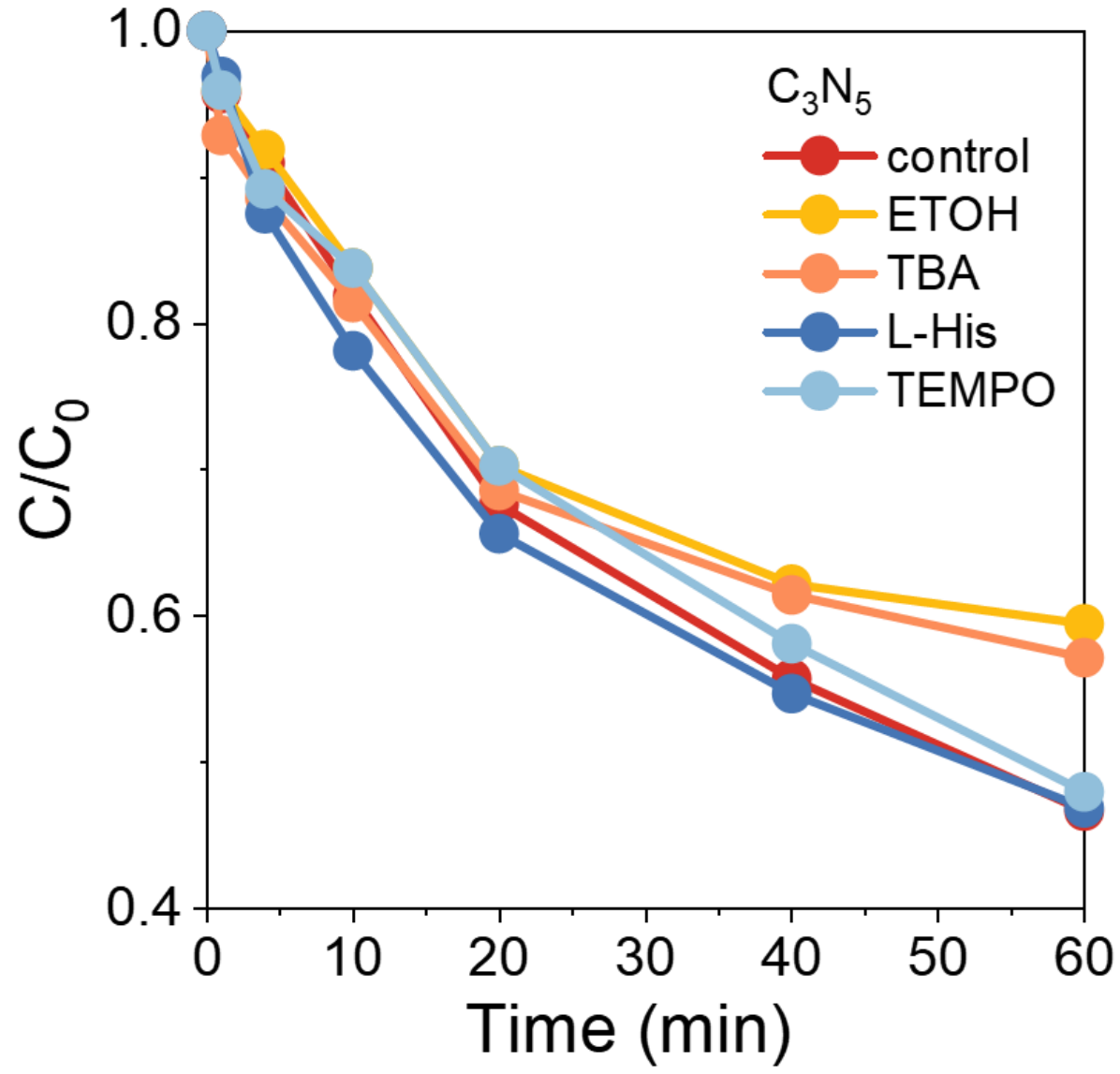

Figure S23. Effects of scavengers on OTC degradation in the C_3N_5.

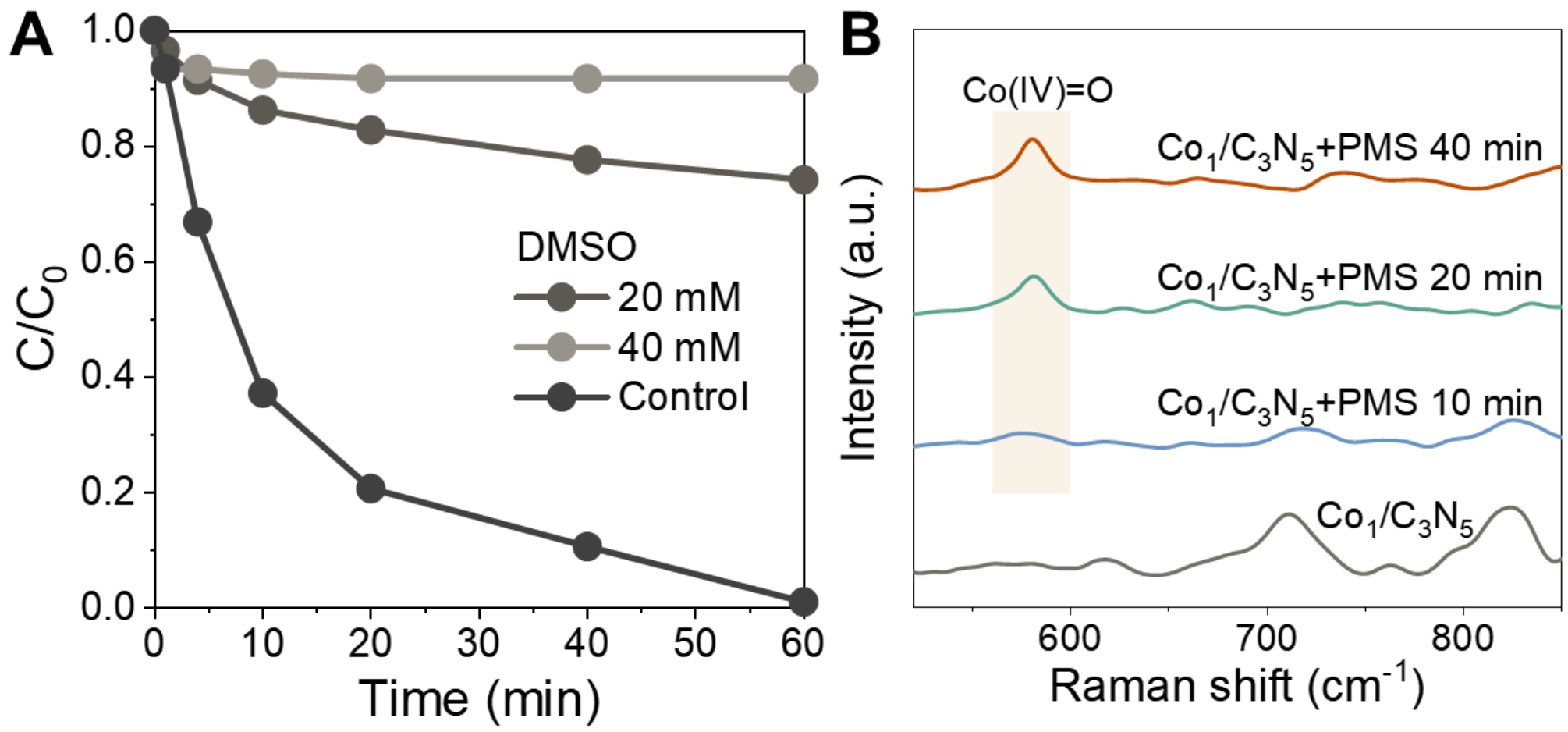

Figure S24. (a) Effects of various dimethyl sulfoxide (DMSO) dosages on OTC degradation in the Co_1/C_3N_5/PMS system. (b) In situ Raman spectra of the Co_1/C_3N_5/PMS system.

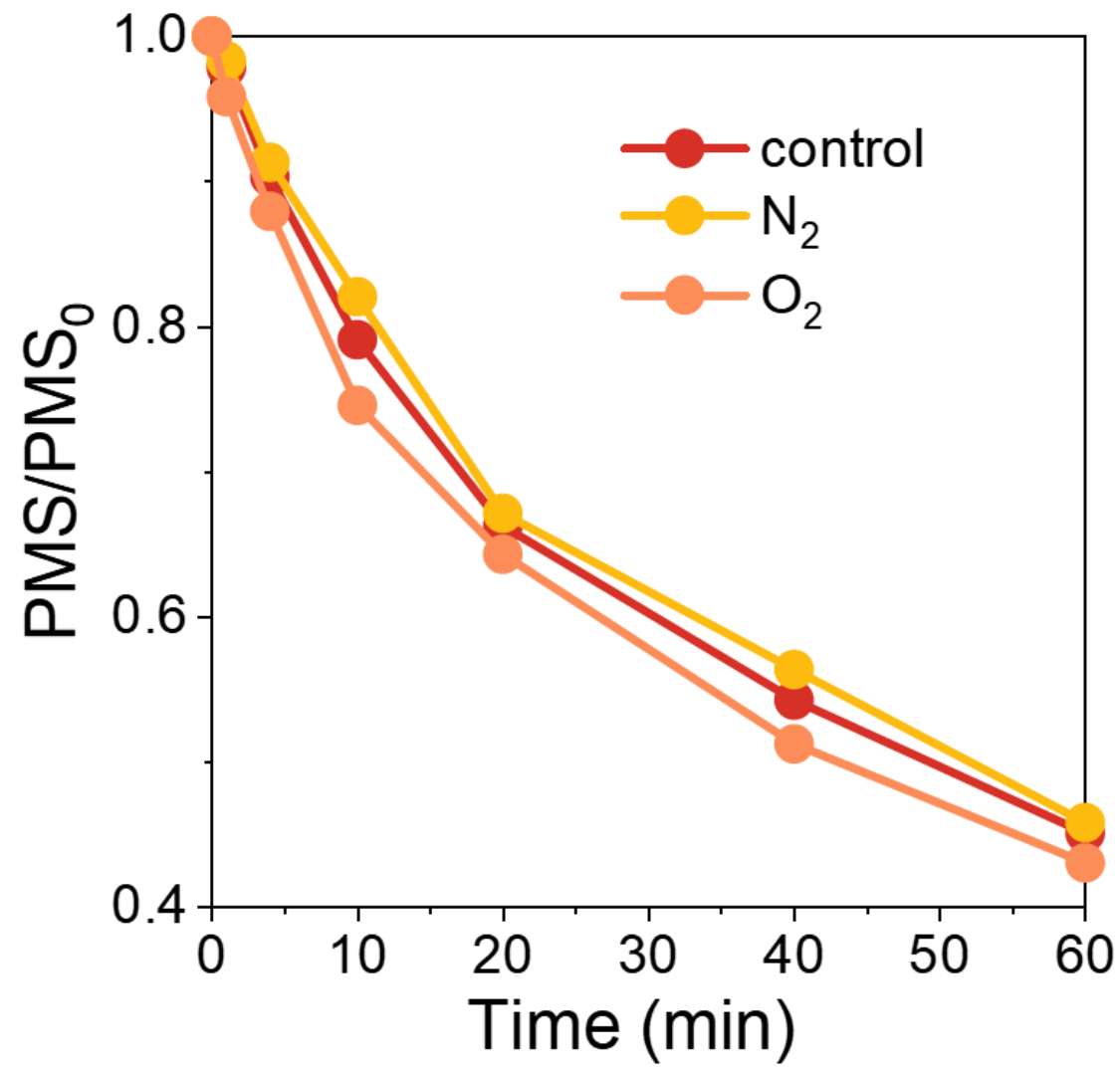

Figure S25. The corresponding PMS consumption in Co_1/C_3N_5/PMS system.

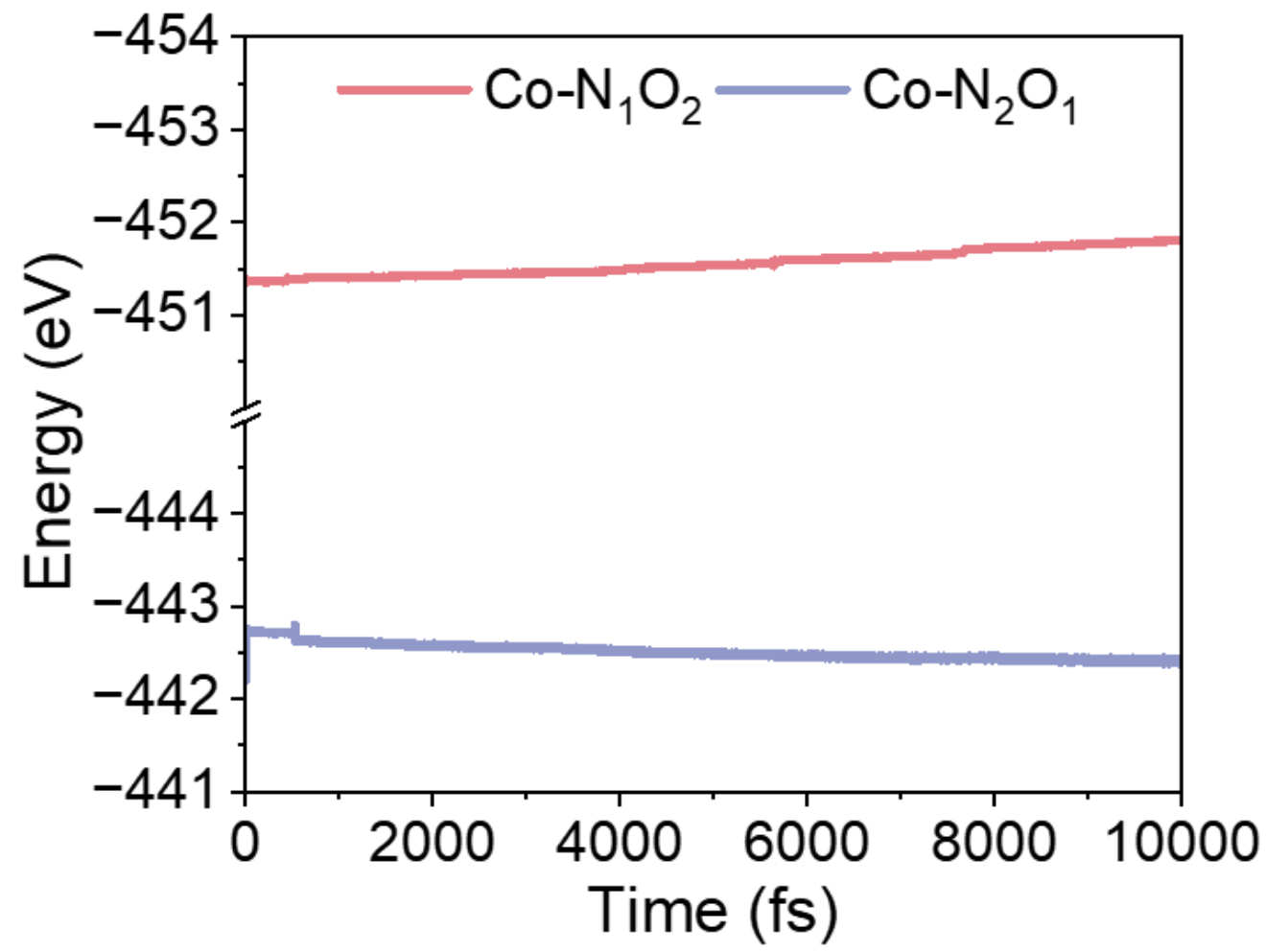

Figure S26. The total energy along the whole 10 ps AIMD sampling periods for Co-N_2O_1 and Co-N_1O_2.

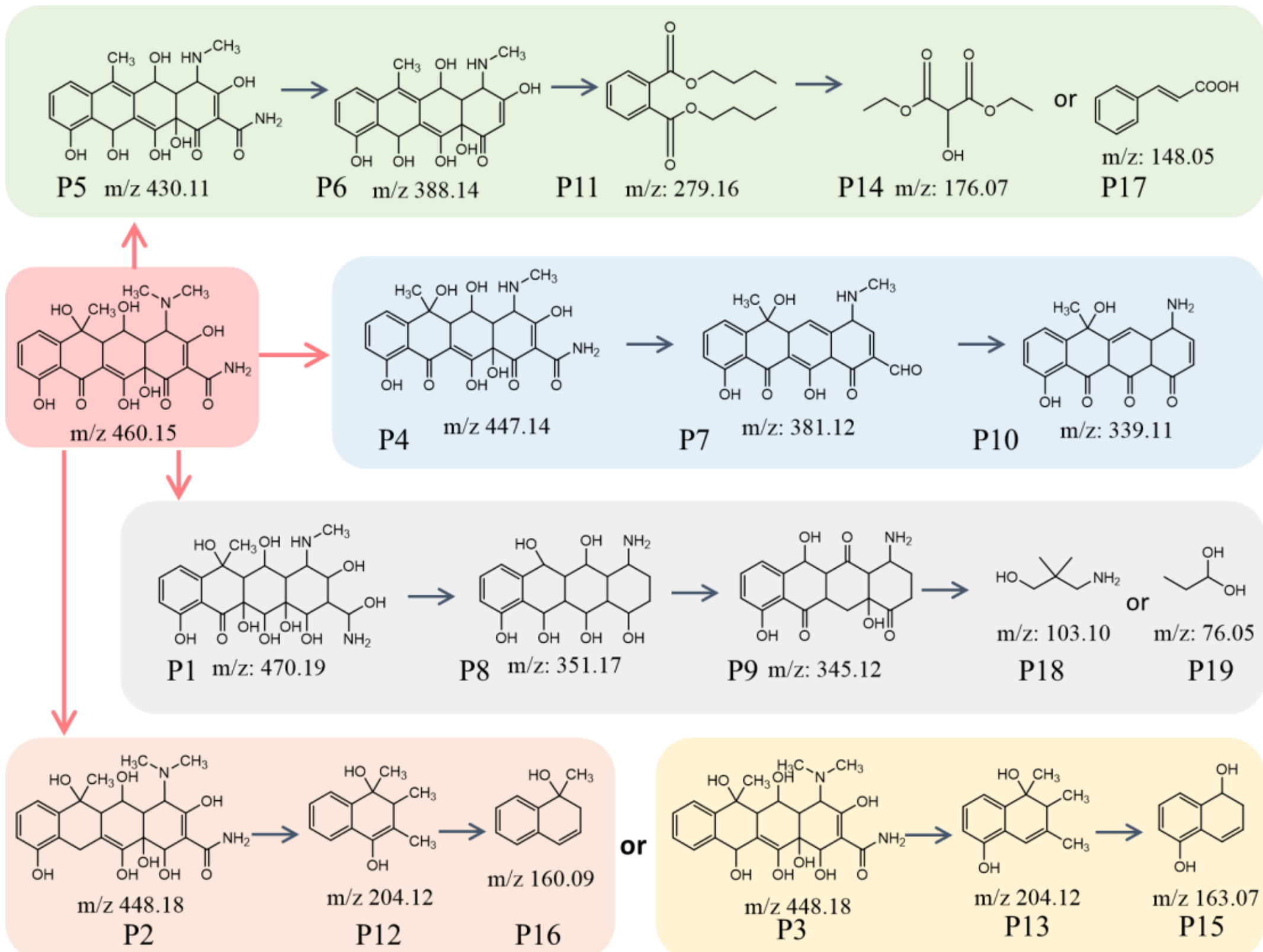

Figure S27. Degradation products and proposed degradation pathways of DCF in the Co_1/C_3N_5 coupled PMS system.

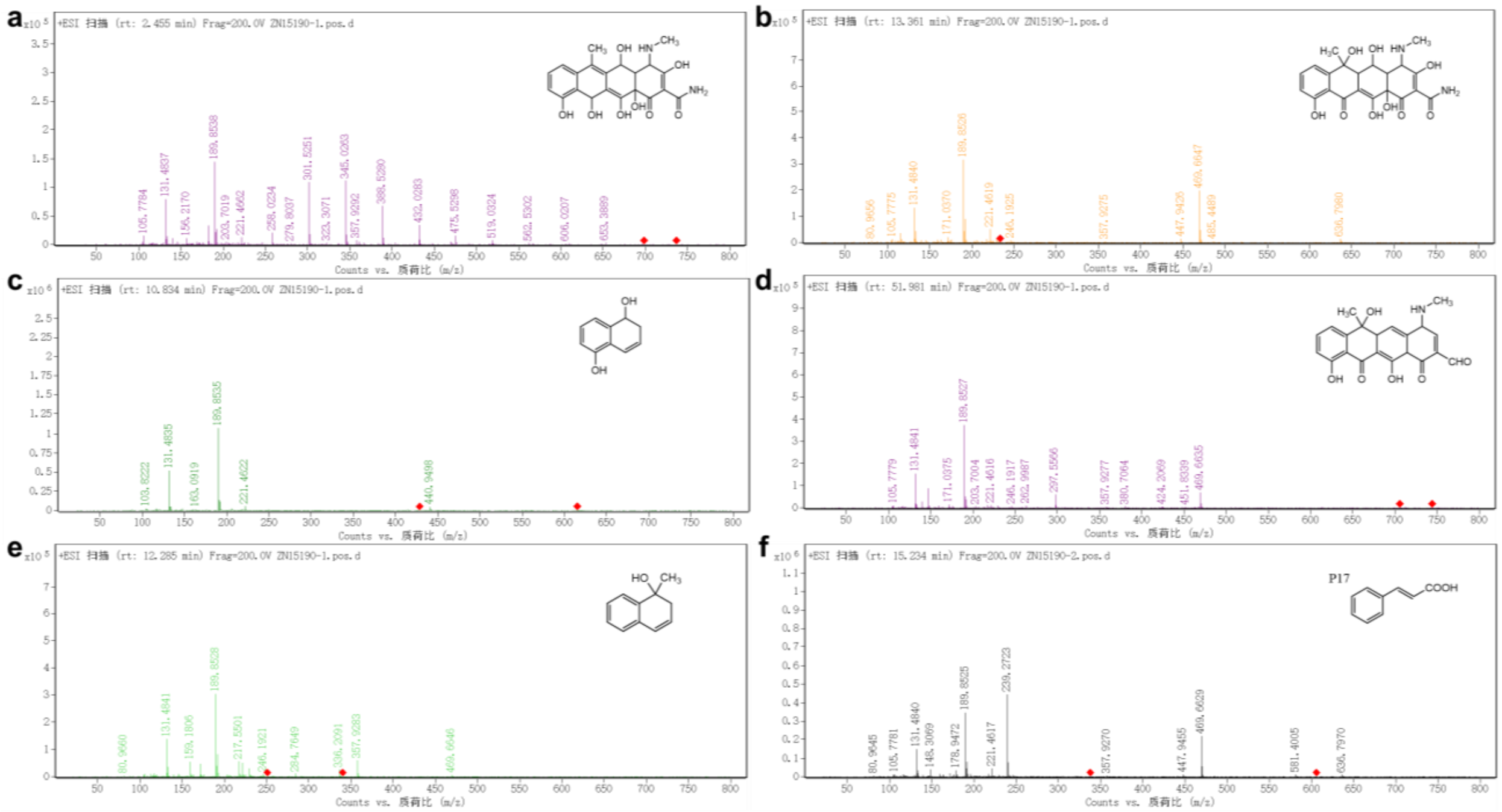

Figure S28. Mass spectra of characteristic intermediates collected.

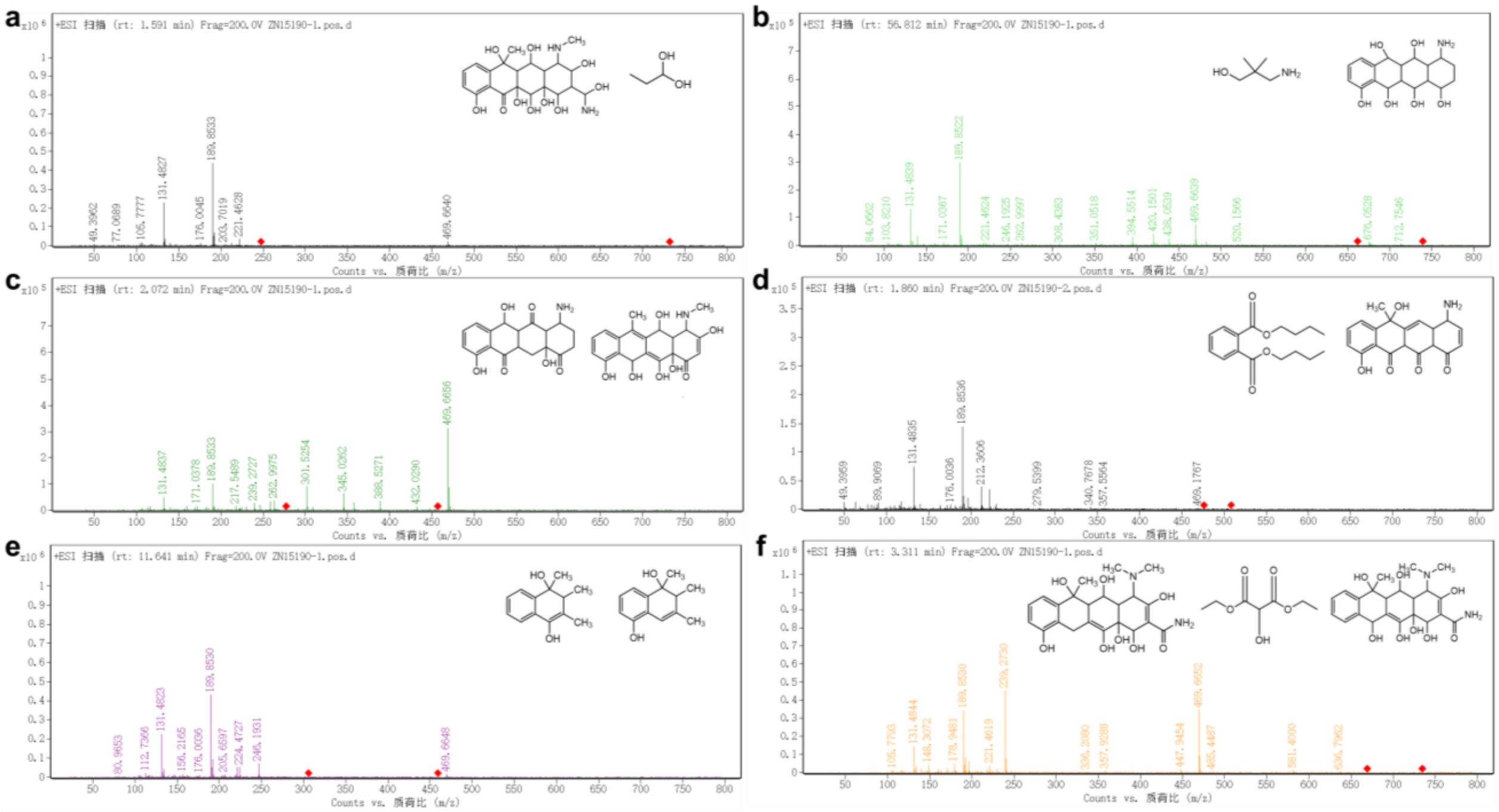

Figure S29. Mass spectra of characteristic intermediates collected.

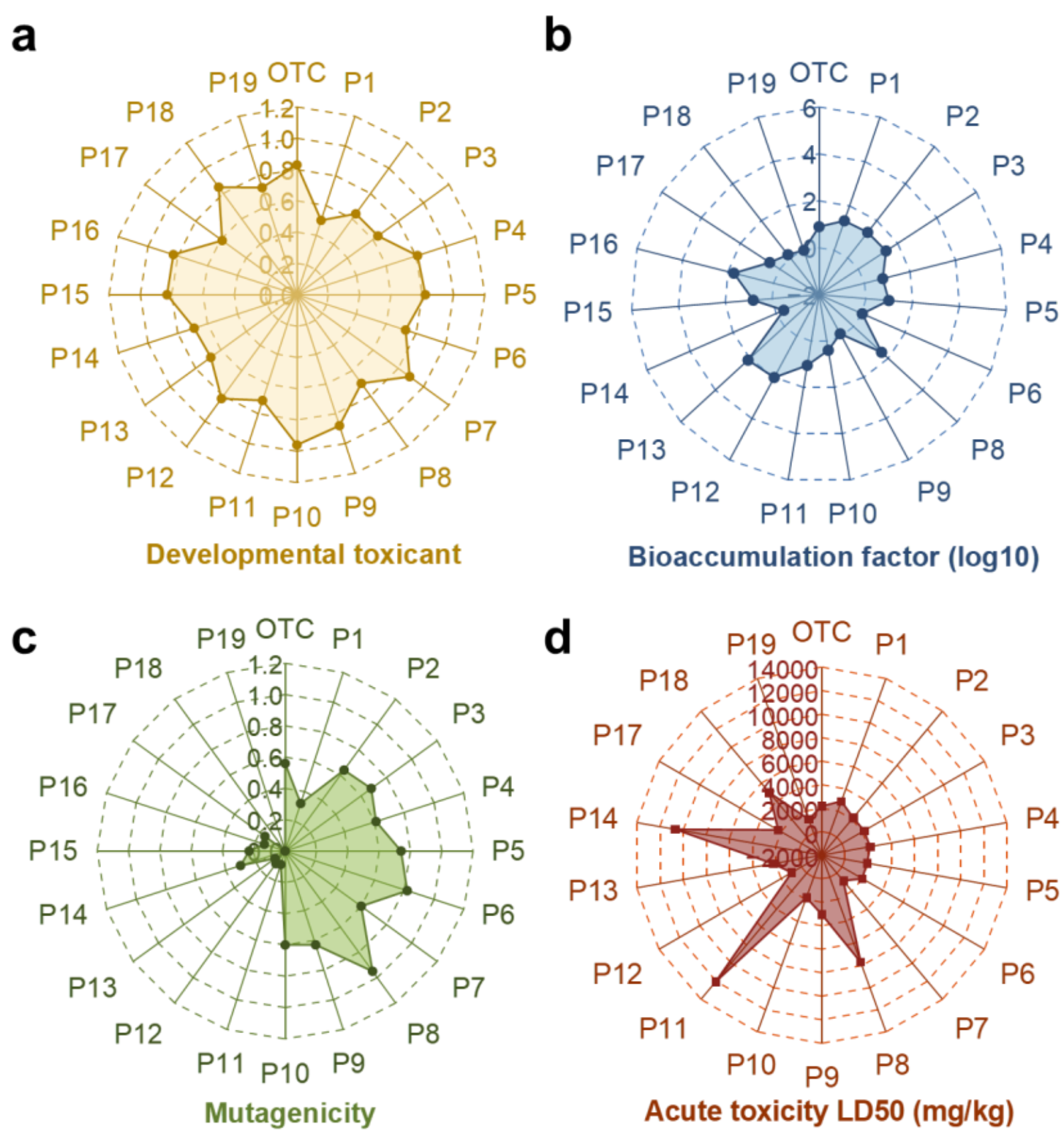

Figure S30. (a) Developmental toxicity, (b) bioaccumulation factor, (c) mutagenicity, and (d) acute toxicity of OTC and their corresponding products in the Co_1/C_3N_5 coupled PMS system.

SUPPLEMENTARY TABLES

Table S1. EXAFS fitting parameters at the Co K-edge for Co_1/C_3N_5.

fresh	path	N	R (Å)	σ^2 (Å^2)	ΔE_0 (eV)	R factor
Co-N_2O_1	Co-N	2.300	1.976	0.008	-0.656	0.014
	Co-O	1.400	2.043	0.009		
Co-N_1O_2	Co-N	0.900	1.981	0.004	-0.173	0.019
	Co-O	2.300	2.120	0.005		
Co-N_3	Co-N	3.200	2.000	0.009	-1.012	0.012

N: coordination number; R: bond distance; σ^2: Debye-Wall factor; ΔE_0: the inner potential correction; R factor: goodness of fit.

Table S2. Comparison of the kinetics of organic contaminant degradation. The modified kinetic rate constant (k-value)* was calculated through dividing the observed rate constant of organic contaminants by the catalyst dosage and oxidant concentration, followed by multiplying organic contaminant.

Catalyst	Catalyst (g/L)	PMS (mM)	pollutant (mg L-')	kobs (min-)	k-value (min^{-1} .M^{-1})	Ref
Co_1/C_3N_5	1.2	0.3249	6	0.1006	1.5477	This work
MIL-88A(Fe)$CoFe_2O_4$	0.25	(PS) 3.6993	10	0.0117	0.1265	[6]
FeCoNC	0.3	2	80	0.0107	1.4267	[7]
CoCNx@SBA-15	0.0375	2.5	9.9012	0.0116	1.2251	[8]
Co-δ-MnO_2	0.1	5	10	0.0098	0.1960	[9]
Co_3O_4/CNTs	0.5263	3.6993	20	0.0334	0.3431	[10]
Ni(II)-citrate	0.554	2.5	20	0.0820	1.1841	[11]
NCT-β	2	1.6247	50	0.0500	0.7694	[12]
Fe_3O_4@β-CD	1	(PS) 5	22.8	0.0252	0.1149	[13]
NiO	0.3	5	9.411	0.1600	1.0038	[14]
DCASNs	1	(H_2O_2) 10	22.8	0.0670	0.1528	[15]
LFO-Cu	1	3.25	25	0.0640	0.4923	[16]
$CuFe_2O_4$	0.2	0.1	5	0.0037	0.9250	[17]
OVc-CNOPNfs	0.8	10	10	0.0690	0.0863	[18]
Ni–N–CNTs	0.1	1	10	0.0003	0.0270	[19]
gC_3N_4/Ni	0.25	0.5	20	0.0019	0.2992	[20]
$Ag_{0.4}$-$BiFeO_3$	0.3	5	10	0.0338	0.2253	[21]
$Cu_{0.2}Ni_{0.8}O$/SBA-15	2	1	14.236	0.0850	0.6050	[22]
Ni@NCNT	0.2	(PS) 7.3986	20	0.0857	1.1582	[23]

CBC	0.8	(PS) 3.6	10	0.0040	0.0139	[24]
N-RGO/Fe_3O_4	0.1	(PS) 10	20	0.0100	0.2000	[25]
$MnFe_2O_4$	0.6	(H_2O_2) 200	10	0.0200	0.0017	[26]
γ-Cu–Al_2O_3	1	(H_2O_2) 10	22.8	0.0237	0.0540	[27]
NMS	0.15	5	20	0.0380	1.0133	[28]
d-g-C_3N_4-Cu	2	(H_2O_2) 10	25	0.0230	0.0288	[29]
CFTS	1	5	22.829	0.1106	0.5050	[30]
Fe/Ni_1@PNBC	2	3.2494	80	0.0400	0.4924	[31]
BBC_2	0.3	2	20	0.0293	0.9767	[32]
α-FeOOH/GBC	0.15	30	10	0.0210	0.0467	[33]
L-Arginine -TiO_2/g-C_3N_4	1	5	50	0.0413	0.4131	[34]
H_3BO_3@$Bi_2Fe_4O_9$	0.2	48.9	20	0.0880	0.1799	[35]
Vo-Fe_3O_4	50	2	10	0.0311	0.0031	[36]
G-Cu	0.2	5	10	0.0717	0.7165	[37]

$$\text{k-value}=\frac{k_{obs}*c[\text{Pollutent}]}{c[\text{Catalyst}]*c[\text{PMS}]}$$

Table S3. EXAFS fitting parameters at the Co K-edge for Co_1/C_3N_5-used.

used	path	N	R (Å)	σ^2 ($Å^2$)	ΔE_0 (eV)	R factor
$Co-N_1O_2$	Co-O	2.300	2.008	0.003	1.839	0.009
	Co-N	1.200	2.191	0.003		
$Co-N_2O_1$	Co-N	2.300	1.963	0.003	1.839	0.018
	Co-O	1.200	2.091	0.003		
$Co-N_3$	Co-N	3.200	2.026	0.007	0.220	0.014

N: coordination number; R: bond distance; σ^2: Debye-Wall factor; ΔE_0: the inner potential correction; R factor: goodness of fit.

Table S4. EXAFS fitting parameters at the Co K-edge for Co_1/C_3N_5-regeneration.

regeneration	path	N	R (Å)	σ^2 (Å^2)	ΔE_0 (eV)	R factor
Co-N_1O_2	Co-O	2.000	2.012	0.001	-2.254	0.006
	Co-N	0.900	1.844	0.001		
Co-N_2O_1	Co-N	1.900	1.971	0.005	1.146	0.009
	Co-O	1.300	2.072	0.003		

N: coordination number; R: bond distance; σ^2: Debye-Wall factor; ΔE_0: the inner potential correction; R factor: goodness of fit.

REFERENCES

1. Kresse G, Hafner J. Ab initio molecular dynamics for liquid metals. *Phys Rev B Condens Matter*. 1993; **47**: 558–561.

2. Kresse G, Furthmuller J. Efficient iterative schemes for ab initio total-energy calculations using a plane-wave basis set. *Phys Rev B Condens Matter*. 1996; **54**: 11169–11186.

3. Perdew JP, Burke K, Ernzerhof M. Generalized Gradient Approximation Made Simple. *Phys Rev Lett*. 1996; **77**: 3865–3868.

4. Blochl PE. Projector augmented-wave method. *Phys Rev B Condens Matter*. 1994; **50**: 17953–17979.

5. Zhang Z-Q, Duan P-J, Bai C-W *et al.* Surface-hydroxylated single-atom catalyst with an isolated Co-O-Zn configuration achieves high selectivity in regulating active species. *Nat Commun*. 2025; **16**: 2376.

6. Tran D-T, Bui T-T-U, Phan A-T *et al.* Boosting tetracycline degradation by integrating MIL-88A (Fe) with $CoFe_2O_4$ persulfate activators. *Environ Technol Innov*. 2024; **33**: 103502.

7. Zhang B, Li X, Bingham PA *et al.* Carbon matrix with atomic dispersion of binary cobalt/iron-N sites as efficient peroxymonosulfate activator for organic pollutant oxidation. *Chem Eng J*. 2023; **451**: 138574.

8. Li X, Zhang W, Liu Z *et al.* Effective removal of tetracycline from water by catalytic peroxymonosulfate oxidation over Co@MoS_2: Catalytic performance and degradation mechanism. *Sep Purif Technol*. 2022; **294**: 121139.

9. Wang Y, Sun Y, Wang R *et al.* Activation of peroxymonosulfate with cobalt embedded in layered δ-MnO_2 for degradation of dimethyl phthalate: Mechanisms, degradation pathway, and DFT calculation. *J Hazard Mater*. 2023; **451**: 130901.

10. Liu D, Li M, Li X *et al.* Core-shell Zn/Co MOFs derived Co_3O_4/CNTs as an efficient magnetic heterogeneous catalyst for persulfate activation and oxytetracycline degradation. *Chem Eng J*. 2020; **387**: 124008.

11. Lu Y, Yang F, Chen S *et al.* Decomplexation of Ni(II)-citrate and recovery of nickel from chelated nickel containing electroplating wastewater by peroxymonosulfate with nickel. *Sep Purif Technol*. 2022; **283**: 120142.

12. Xiang Y, Lan J, Cai Y *et al.* Modified nickel–cobalt tailing activated PMS for highly efficient degradation of organic pollutants: Multiple-scale effect and active sites. *Chem Eng J*. 2024; **482**: 149196.

13. Zhu Y, Yue M, Natarajan V *et al.* Efficient activation of persulfate by Fe_3O_4@β-cyclodextrin nanocomposite for removal of bisphenol A. *RSC Adv*. 2018; **8**: 14879–14887.

14. Oh H, Kim J-Y, Chae KH *et al.* Oxyanion-Sensitive Catalytic Activity of Ni(II)/Oxyanion Systems for Heterogeneous Organic Degradation: Differential Oxidizing Capacity of Ni(III) and Ni(IV) as High-Valent Intermediates. *Environ Sci Technol*. 2024; **58**: 16642–16655.

15. Lyu L, Zhang L, Hu C *et al.* Enhanced Fenton-catalytic efficiency by highly accessible active sites on dandelion-like copper–aluminum–silica nanospheres for water purification. *J Mater Chem A*. 2016; **4**: 8610–8619.

16. Guan Y, Zhou G, Li L *et al.* Cu-doped oxygen-rich vacancy MOFs derived perovskite for enhanced mineralization of refractory organics through synergistic non-radical species effects. *Sep Purif Technol*. 2024; **335**: 126072.

17. Deng J, Xu M, Qiu C *et al.* Magnetic $MnFe_2O_4$ activated peroxymonosulfate processes for degradation of bisphenol A: Performance, mechanism and application feasibility. *Appl Surf Sci*. 2018; **459**: 138–147.

18. Cao W, Lyu L, Deng K *et al.* l-Ascorbic acid oxygen-induced micro-electronic fields over metal-free polyimide for peroxymonosulfate activation to realize efficient multi-pathway destruction of contaminants. *J Mater Chem A*. 2020; **8**: 810–819.

19. Miao J, Zhu Y, Lang J *et al.* Spin-State-Dependent Peroxymonosulfate Activation of Single-Atom M-N Moieties via a Radical-Free Pathway. *ACS Catal*. 2021; **11**: 9569–9577.

20. Tian M, Ren X, Ding S *et al.* Effective degradation of phenol by activating PMS with bimetallic Mo and Ni Co-doped g C_3N_4 composite catalyst: A Fenton-like degradation process promoted by non-free radical $^1O_2$. *Environ Res*. 2024; **243**: 117848.

21. Ouyang M, Li X, Xu Q *et al.* Heterogeneous activation of persulfate by Ag doped $BiFeO_3$ composites for tetracycline degradation. *J Colloid Interface Sci*. 2020; **566**: 33–45.

22. Yu D, He J, Xie T *et al.* Peroxymonosulfate activation using a composite of copper and nickel oxide coated on SBA-15 for the removal of sulfonamide antibiotics. *Environ Res*. 2022; **206**: 112301.

23. Kang J, Duan X, Wang C *et al.* Nitrogen-doped bamboo-like carbon nanotubes with Ni encapsulation for persulfate activation to remove emerging contaminants with excellent catalytic stability. *Chem Eng J*. 2018; **332**: 398–408.

24. Wang B, Li Y-n, Wang L. Metal-free activation of persulfates by corn stalk biochar for the degradation of antibiotic norfloxacin: Activation factors and degradation mechanism. *Chemosphere*. 2019; **237**: 124454.

25. Peng G, Zhang M, Deng S *et al.* Adsorption and catalytic oxidation of pharmaceuticals by nitrogen-doped reduced graphene oxide/Fe_3O_4 nanocomposite. *Chem Eng J*. 2018; **341**: 361–370.

26. Wang G, Zhao D, Kou F *et al.* Removal of norfloxacin by surface Fenton system ($MnFe_2O_4/H_2O_2$): Kinetics, mechanism and degradation pathway. *Chem Eng J*. 2018; **351**: 747–755.

27. Lyu L, Zhang L, Wang Q *et al.* Enhanced Fenton Catalytic Efficiency of γ-Cu–Al_2O_3 by σ-Cu^{2+}–Ligand Complexes from Aromatic Pollutant Degradation. *Environ Sci Technol*. 2015; **49**: 8639–8647.

28. Song X, Diao S, He W *et al.* Design of active dual atom Ni-Co-2H-MoS_2 catalyst: Synergistic effect of Ni-adsorption and co-catalysis for activating peroxymonosulfate. *Sep Purif Technol*. 2024; **333**: 125927.

29. Jiang N, Lyu L, Yu G *et al.* A dual-reaction-center Fenton-like process on –C=N–Cu linkage between copper oxides and defect-containing g-C_3N_4 for efficient removal of organic pollutants. *J Mater Chem A*. 2018; **6**: 17819–17828.

30. Kong L, Fang G, Chen Y *et al.* Efficient activation of persulfate decomposition by Cu_2FeSnS_4 nanomaterial for bisphenol A degradation: Kinetics, performance and mechanism studies. *Appl Catal B-Environ*. 2019; **253**: 278–285.

31. Chen Y, Liu M, Fan W *et al.* Pine needle biochar loaded with iron-nickel bimetal composite as heterogeneous catalysts to activate peroxymonosulfate for efficient degradation of oxytetracycline. *Desalin Water Treat*. 2023; **282**: 220–236.

32. Gao S, Wang Z, Wang H *et al.* Peroxydisulfate activation using B-doped biochar for the degradation of oxytetracycline in water. *Appl Surf Sci*. 2022; **599**: 153917.

33. Chen Z, Lai C, Qin L *et al.* Synergy between graphitized biochar and goethite driving efficient H_2O_2 activation: Enhanced performance and mechanism analysis. *Sep Purif Technol*. 2023; **314**: 123516.

34. Eskandari P, Amarloo E, Zangeneh H *et al.* Photocatalytic degradation of metronidazole and oxytetracycline by novel l-Arginine (C, N codoped)-TiO_2/g-C_3N_4: RSM optimization, photodegradation mechanism, biodegradability evaluation. *Chemosphere*. 2023; **337**: 139282.

35. Dong J, Zou W, Liu G *et al.* Surface boric acid modification promoted Fe^{3+}/Fe^{2+} cycle and H_2O_2 activation for facilitating Fenton degradation performance of $Bi_2Fe_4O_9$. *Colloids Surf A*. 2023; **678**: 132477.

36. Li L, Cheng M, Almatrafi E *et al.* Tuning the intrinsic catalytic sites of magnetite to concurrently enhance the reduction of H_2O_2 and O_2: Mechanism analysis and application potential evaluation. *J Hazard Mater*. 2023; **457**: 131800.

37. Lai C, Yang L, Li L *et al.* Design of a highly efficient Cu-based catalyst with two functional areas: The role of Cu^0 and oxygen vacancies in Fenton-like system. *Chem Eng J*. 2023; **464**: 142420.
